# Methods for quantification of growth and productivity in anaerobic microbiology and biotechnology

**DOI:** 10.1007/s12223-018-0658-4

**Published:** 2018-11-16

**Authors:** Lisa-Maria Mauerhofer, Patricia Pappenreiter, Christian Paulik, Arne H. Seifert, Sébastien Bernacchi, Simon K.-M. R. Rittmann

**Affiliations:** 1 Archaea Physiology & Biotechnology Group, Archaea Biology and Ecogenomics Division, Department of Ecogenomics and Systems Biology, Universität Wien, Althanstraße 14, 1090 Wien, Austria; 2 Institute for Chemical Technology of Organic Materials, Johannes Kepler University Linz, Linz, Austria; 3 Krajete GmbH, Linz, Austria

**Keywords:** Archaea, Bacteria, Physiology, Microscopy, Molecular biology, Process analytical technology, Bioprocess, Cultivation

## Abstract

Anaerobic microorganisms (anaerobes) possess a fascinating metabolic versatility. This characteristic makes anaerobes interesting candidates for physiological studies and utilizable as microbial cell factories. To investigate the physiological characteristics of an anaerobic microbial population, yield, productivity, specific growth rate, biomass production, substrate uptake, and product formation are regarded as essential variables. The determination of those variables in distinct cultivation systems may be achieved by using different techniques for sampling, measuring of growth, substrate uptake, and product formation kinetics. In this review, a comprehensive overview of methods is presented, and the applicability is discussed in the frame of anaerobic microbiology and biotechnology.

## Introduction

Anaerobic microorganisms are widespread in almost all environments on Earth. They are natural inhabitants of anaerobic ecological niches such as aqueous sediments of rivers, lakes, and oceans, sediments of soils, and the gastrointestinal tract of animals. Their energy metabolism is adapted to a molecular oxygen (O_2_)-free environment. In such environments, substrate-limiting conditions are often encountered (Lever et al. 2015). To gain energy and/or carbon, some anaerobes degrade organic matter, e.g., lignocellulose, polysaccharides, proteins, or lipids. Other anaerobes metabolize short chain fatty acids, volatile fatty acids (VFAs), and alcohols while some also possess a streamlined and efficient physiology for performing a biological gas-to-product conversion. Those metabolic processes are known to play an important role in the global carbon cycle (Bond and Templeton 2011; Rumpel and Kögel-Knabner 2011; Schmidt et al. 2011; Hatti-Kaul and Mattiasson 2016).

Substrates for biotechnological production processes are available in a wide range as solids (e.g., biomass, ore), liquids, or gases. Solid substrates are commonly used in biogas plants in the form of energy crops (e.g., maize) or as agricultural wastes (Amon et al. 2007; Bond and Templeton 2011). Those solid substrates can be converted by a consortium of different microbes to either liquid or gaseous products, which could be further metabolized to VFAs and gases (Bond and Templeton 2011).

Solid biomass is degraded through a microbial process chain, referred to as hydrolysis, where extracellular enzymes break down complex carbohydrates, proteins, and lipids into their basic constituents. The generated constituents serve as products for acidogenesis or acetogenesis as well as for molecular hydrogen (H_2_) and carbon dioxide (CO_2_) production. Those reactions are supported by facultative anaerobic bacteria, which metabolize residual O_2_ in anaerobic digesters, thereby establishing suitable conditions for the final step in the anaerobic food chain, which is referred as biological methane (CH_4_) production mediated by obligate anaerobic archaea. This process, of solid biological raw material or waste processing, results in a production of biogas containing approximately 50–70 vol.% CH_4_, 30–50 vol.% CO_2_, and small amounts of other gases, e.g. hydrogen sulfide (H_2_S) (Sasse 1988). The final exhaust gas composition depends on the applied substrates. The main product generated from anaerobic digestion is CH_4_, whereas CO_2_ is regarded as by-product.

Liquid substrates that are used in anaerobic microbiology and biotechnology are organic acids, glycerol, and sugars. One of the highly relevant organic acids in biotechnology is formate (Kim et al. 2010; Rittmann et al. 2015a; Kottenhahn et al. 2018; Ergal et al. 2018). Formate can be produced from carbon monoxide (CO) that is generated as a byproduct through the Linz–Donawitz manufacturing process (Atwater 1942). Formate is a highly suitable substrate for H_2_ production by archaea (Bae et al. 2012, 2015). Glycerol is considered as an important biotechnologically relevant substrate due to the fact that it is produced as a by-product from the biodiesel manufacturing process. Currently, there are already many bioprocesses that utilize glycerol for production of citric acid, lactic acid, 1,3-dihydroxyacetone (DHA), 1,3-propanediol (1,3-PD), dichloro-2-propanol (DCP), acrolein, H_2_, ethanol, etc. (Fan et al. 2010). Moreover, sugars can be utilized as substrate for microbial production of acetone–butanol–ethanol (Friedl et al. 1991; Kujawska et al. 2015) or microbial H_2_ production (Rittmann and Herwig 2012; Rittmann et al. 2015a; Reischl et al. 2018a; Ergal et al. 2018). Another well-established anaerobic process that utilizes pure cultures is the anaerobic ammonium oxidation (ANAMOX) process (Innerebner et al. 2007; Ali and Okabe 2015). In the ANAMMOX process, ammonium and nitrite are comproportionated to molecular nitrogen (N_2_). This process has already reached commercial scale.

CO, H_2_, CO_2_, and CH_4_ are gaseous substrates or products that can respectively be utilized in anaerobic microbiology and biotechnology by carboxydotrophic, hydrogenotrophic, autotrophic, or methanotrophic microorganisms. However, until now no pure culture of an anaerobic methanotrophic microorganism was isolated. Anaerobic microbial growth on, e.g., CO or H_2_/CO_2_ using a pure culture of microorganisms in a biological gas-to-gas conversion processes is well known (Bae et al. 2012; Rittmann et al. 2015b). These processes are efficiently performed with archaea and even highly competitive compared to chemical gas to gas conversion processes (Bernacchi et al. 2014a, 2014b). A hallmark of such processes is that even by-product CO_2_ from the anaerobic digestion process can be upgraded to CH_4_ through an ex situ CO_2_-based biological methane production (CO_2_-BMP) process that can be performed with methanogens (Seifert et al. 2013; Rittmann 2015; Rittmann et al. 2015a, b; Rachbauer et al. 2016). Moreover, it was shown that CO_2_ emission from flue gases can be converted to CH_4_ by *Methanothermobacter marburgensis* (Seifert et al. 2013). The CO_2_-BMP technology could also be integrated in various other CO_2_ utilization scenarios where biological gas-to-gas conversion processes could be utilized (Martínez-Porqueras et al. 2012; Rachbauer et al. 2016; Abdel Azim et al. 2017). The aforementioned biological gas-to-gas conversion processes have already reached commercial plant scale.

To assess the role of anaerobic microorganisms under natural growth conditions and to be able to investigate their metabolic capabilities and their physiological potential, cultivation is inevitable. Among others, cultivation of microbes allows investigating physiological responses (Valentine et al. 1994), the metabolism (Ghose et al. 1978), and the interaction with potential syntrophic partners (Shen et al. 2016). Depending on the organism of interest, different micro- and macro-nutrients for sustaining and improving growth and/or product formation are required. Therefore, it is of great interest to increase the amount of viable cells in a population and/or to optimize cultivation conditions to reach high productivities and/or yields.

**Fig. 1:**
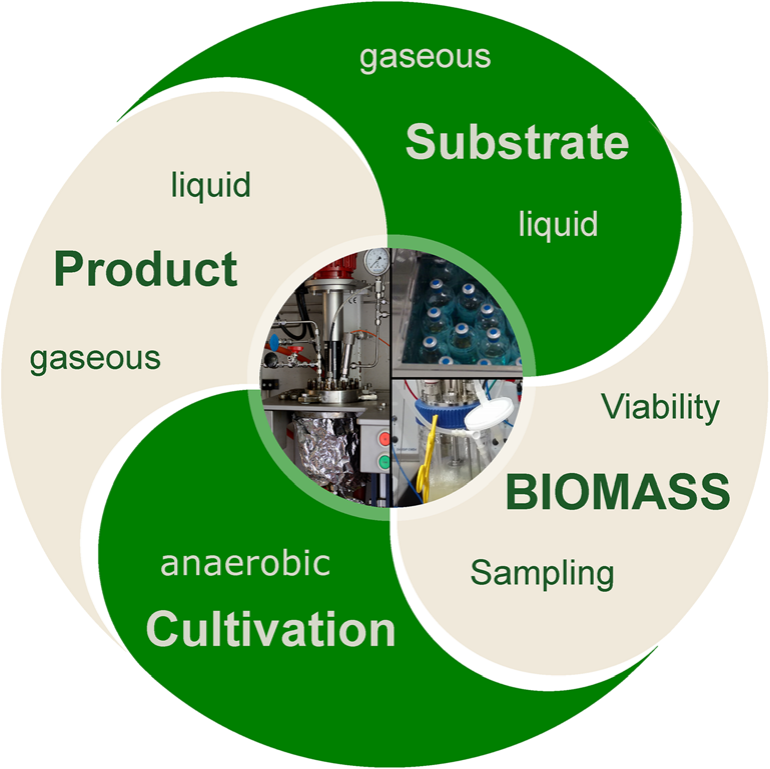
Overview of the following review, summarizing important topics (anaerobic cultivation, biomass sampling, biomass concentration and viability, identification and quantification of liquid and gaseous substrates and products)

This review provides an overview on offline, at-line, and online methods that are currently applied in anaerobic microbiology and biotechnology for quantification of solid (e.g., biomass), liquid, and gas production. In the first part of the review, anaerobic cultivation techniques for the creation of an anoxic atmosphere for cultivation of anaerobes as well as proper cultivation vessels and sampling methods will be discussed. The second part of the review will present techniques that can be used to monitor or quantify microbial growth, population activity, substrate(s) uptake, and product(s) formation kinetics in anaerobic microbial systems consisting of microbial pure or defined co-cultures. Finally, the applicability of these methods is discussed from an ecological to a bioprocess technological point of view with a special emphasis on, but not limited to, anaerobic and axenic cultures (Fig. 1).

## Anaerobic microbiology, and biomass cultivation techniques

The target of most cultivations using microorganisms is the propagation of cells for the purpose of examining, e.g., morphological, physiological, and biotechnological characteristics by increasing the number of cells or a population of cells in a specific cell cycle stage. The increase of cells within a certain time period implies the assimilation of macro-nutrients like such as carbon containing substrate(s) and, e.g., phosphorous, N_2_, containing compounds allowing the last step of microbial reproduction: cell division. Before cells can divide, DNA has to be replicated, the daughter cell has to be constructed, and finally cell division is being induced. A microbial life span is characterized by different stages, referred as cell cycle, which can be divided into several stages (Bernander 2000; Lindås et al. 2008; Zaritsky and Woldringh 2015; den Blaauwen et al. 2017). In physiology and biotechnology, tracing how fast substrates are taken up and converted into a product is critical when assessing the metabolic efficiency of a microorganism. Each microorganism possesses a specific substrate requirement for maintaining its cellular activity. By consuming substrate(s), microorganisms are able to divide at a specific frequency or grow at a specific growth rate (*μ*) and the multivariate relation existing between them is referred to as growth kinetics. The latter implies existence of a consumption rate, at which the substrate (*s*) is utilized within a certain time (*t*), referred to as specific substrate consumption rate (*q*). Growth and substrate utilization can be linearly linked to the yield coefficient (*Y*_*x*/*s*_). This coefficient relates the conversion efficiency of a growth substrate into biomass (*X*) to the specific growth rate (*μ*) and *q*, see Eq. 1 (Monod 1958; Kovarova-Kovar and Egli 1998).

1 $$ \mu =\frac{Y_{X/S}}{X}\cdot \frac{\mathrm{d}s}{\mathrm{d}t}\cong {Y}_{X/S}\cdot q $$

Growth of microorganisms in closed batch, batch, or fed-batch cultivation mode follows different growth phases: lag phase, exponential phase, stationary phase, and death phase, with transition phases in between. Initially, microbes have to adapt to the present condition in the medium (lag phase). Depending on the microbial strain, the lag phase can vary or either be skipped until the exponential growth stage is reached (Stieglmeier et al. 2014). The exponential growth phase commences after the population exits the transition phase that follows after the lag phase. During the exponential growth phase, the microorganisms experience balanced growth. Balanced growth refers to the phenomenon that the population grows at a given/set/controlled *μ*. If the cell density of microbes in the liquid phase reaches a certain concentration, which is sometimes associated with the secretion of quorum sensing molecules, cell division ceases. A further increase of the biomass density can also be terminated if carbon or another nutrient is limiting propagation. Then, the entire population enters a stationary phase. The latter phase is illustrated through an equilibrium between dividing and dying cells. The last stage in the cell growth cycle is the death phase, which is also an exponential function (Koch 2007). A population of microorganisms show a characteristic growth pattern, when inoculated into a fresh culture medium, which could vary when testing different cultivation systems.

### Specificities of anaerobic cultivation

**Fig. 2:**
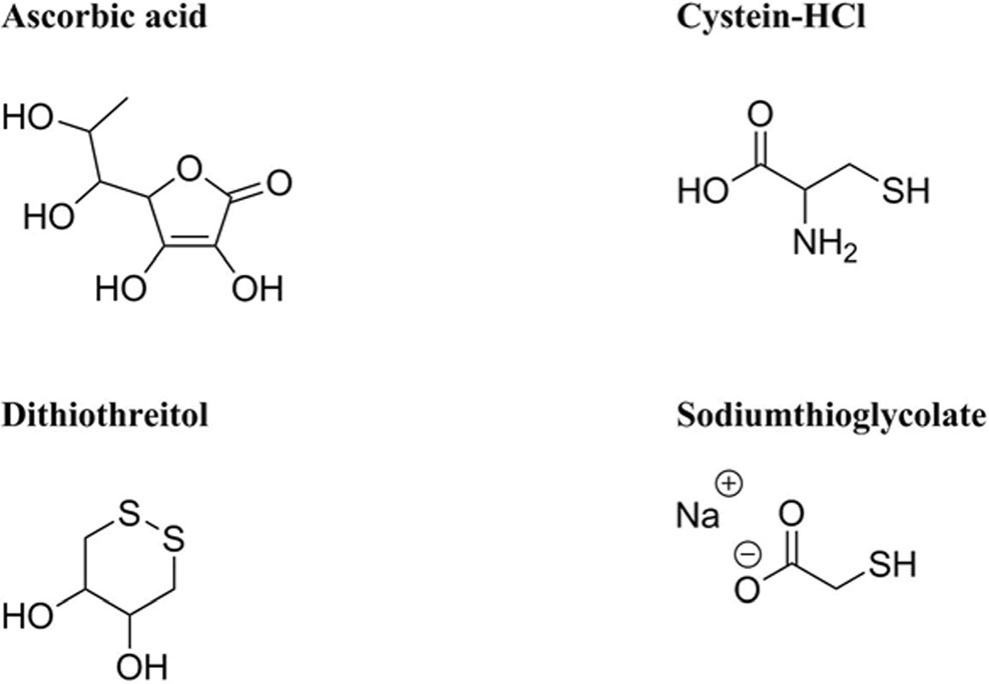
Chemical structures of selected reducing agents

**Fig. 3:**
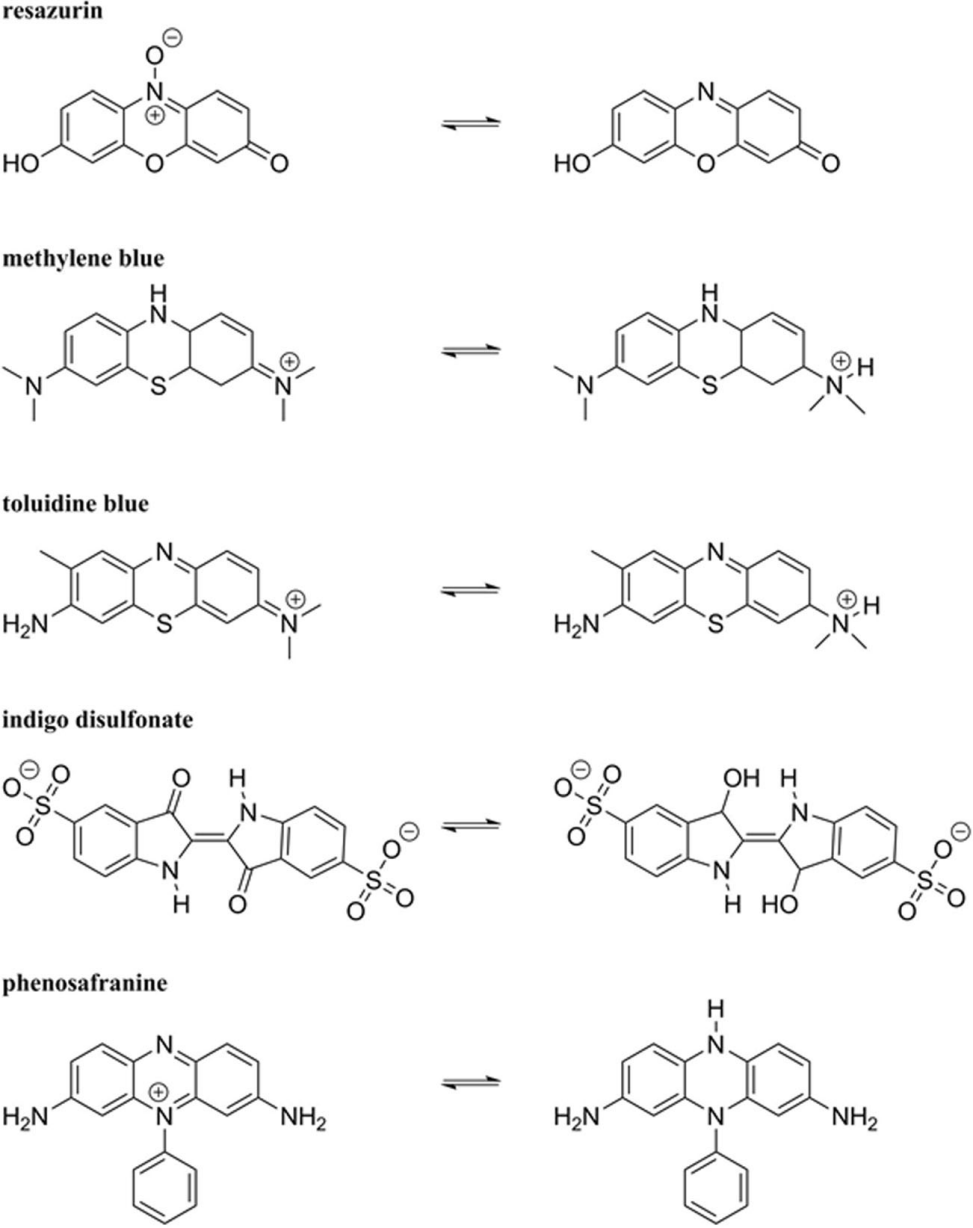
Oxidized (left) and reduced (right) form of the redox dyes. The structure of titanium(III)citrate is not shown due to different forms of the oxidized form depending on the predominant pH in the medium (Collins et al. 2005)

Cultivation of anaerobes may be performed to propagate the microorganism of interest and to produce metabolic end products. Anaerobic microorganism can be found in a wide range of environments (Börner 2016). An organism can be classified with respect to the energy source, the electron donor species/compound and the carbon source it uses. Energy can be either generated through light (photo), or an oxidation–reduction (redox) reaction (chemo). The electron donors can derive from an organic (organo) or inorganic (litho) compound. While carbon sources can either be based on organic (hetero) or inorganic (auto) matter (Madigan et al. 2012). To enrich novel species or to optimize growth and productivity of a given anaerobic strain, specific parameters related to their natural habitat, e.g. a low oxidation–reduction potential (ORP), temperature, pH, and salt concentration, have to be mimicked. The first step in anaerobic cultivation is the application of an anoxic atmosphere. The variable that is commonly used to measure the degree of anaerobiosis is ORP, which was found to differ for aerobic and anaerobic cultures. The ORP value in aerobic cultures is higher compared to anaerobic cultures, since O_2_ acts as an oxidizing agent and therefore increases the ORP value. For anaerobes it is important to control the ORP, ≤ − 100 mV for obligate anaerobes (Breznak and Costilow 2007) and < − 330 mV for strict anaerobes (Hungate 1969), such as methanogenic archaea (methanogens). High ORP (tremendously above the optimal condition) do not stringently kill anaerobes, but growth of certain microorganisms might be impaired (Song et al. 2011), and hence, a proper ORP in the media has to be implemented when culturing microorganisms. The application of reducing agents leads to a decline of the ORP by reducing the residual molecular O_2_ in the medium. Reducing agents are compounds that donate electrons to another chemical substance in a redox reaction. The tendency of substances to either function as an electron donor or electron acceptor is expressed as their standard redox potential (Madigan et al. 2012). The standard ORP of a compound is measured under standard conditions with a standard reference half-cell (H_2_ electrode) (de Bolster 1997). The most commonly used reducing agents, their suggested concentration ranges in media, and their standard ORP are listed in Table 1. The majority of used reducing agents for anaerobic cultivations contain sulfur in the form of sulfide (S^2−^), bisulfide (HS–), or thiole (R–SH). In case, sulfur is a growth inhibitor for a given microorganism, other agents such as titanium(III)citrate or ascorbic acid can be employed, see chemical structures in Fig. 2 (Jones and Pickard 1980). Reducing agents are supposed to be prepared under anoxic conditions as stock solutions and then stored using inert gas in the bottle headspace (Bast 2001a; Breznak and Costilow 2007). Since some reducing agents are known to be physiologically toxic at certain concentrations their use must be carefully evaluated (e.g., *Clostridium botulinum* type E, sodium thioglycolate ≤ 0.01 vol.%, inhibition of growth) (Smith and Pierson 1979). To visually determine the ORP in culture media different redox indicators may be employed. Redox indicators (Table 2) are reacting dyes, which becomes obvious when comparing the color of the oxidized compound to the reduced form in a solution. The color change of the ORP differs for every redox dye. The oxidized and reduced forms of the mentioned dyes are shown in Fig. 3. One of the most widely used redox dyes is resazurin, because of its low toxicity toward microorganism and its high effectiveness even at low concentrations in the range of 1 to 2 μg mL^−1^ (Breznak and Costilow 2007). Titanium(III)citrate can be used as a reducing agent as well as a redox dye since it becomes colorless upon complete oxidation (Zehnder and Wuhrmann 1976). For the cultivation of strict anaerobes phenosafranine and titanium(III)citrate can be employed due to the low standard ORP of the reduced form (OPR_red_) (Bast 2001a). To determine the ORP in bioreactors, online redox probe measurements can be applied (Seifert et al. 2013).

**Table 1:** Commonly used reducing agents in anaerobic microbiology

Reducing agent	Concentration in media	ORP (mV)	Reference
Na_2_S∙9H_2_O	0.025–0.05%	− 571	(Bast 2001a; Breznak and Costilow 2007)
Cysteine-HCl	0.025–0.05%	− 340	(Bast 2001a; Breznak and Costilow 2007)
Dithiothreitol	0.01–0.05%	− 330	(Cleland 2002; Breznak and Costilow 2007)
FeS (amorphous hydrated)	4 μg mL^−1^	− 270	(Brock and Od’ea 1977)
Sodium thioglycolate	0.05–0.1%	− 140	(Bast 2001a; Breznak and Costilow 2007)
Ascorbic acid	0.05–0.1%	+ 58	(Bast 2001a; Breznak and Costilow 2007)
H_2_ (PdCl_2_)	Variable	− 413	(Breznak and Costilow 2007)
Titanium(III)citrate	1–4 mM	− 480	(Zehnder and Wuhrmann 1976; Jones and Pickard 1980)

**Table 2:**
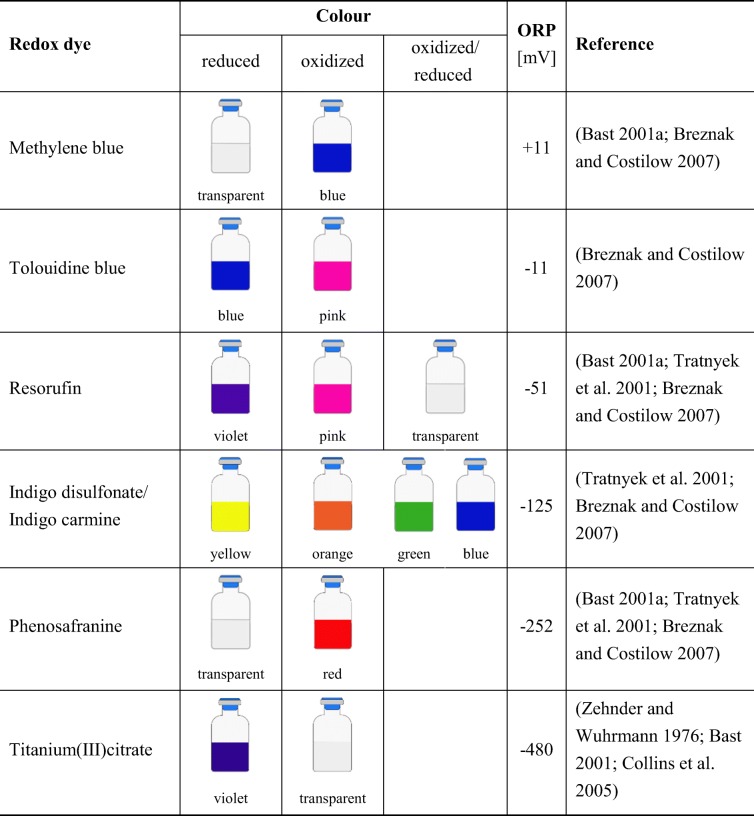
Redox dyes and their corresponding standard ORP values at 30 °C and pH 7.0

The generation of an O_2_-free atmosphere can be achieved by using modified cultivation techniques originally developed by Robert Hungate (Hungate 1969; Balch et al. 1979). The principles of the Hungate technique are briefly summarized as follows: the removal of O_2_ from the medium, mimicking the environmental conditions of the original microbial habitat (composition of the medium, pH, ORP) with a minimized O_2_ exposure during inoculation and a rapid consolidation of the agar with cold water (Hungate 1950). The Hungate technique has been further improved by using pre-reduced anaerobic sterilized media (Moore 1966), butyl rubber stopper for the plugging of the tube (Hungate et al. 1966), crimp closed aluminum seals (Miller and Wolin 1974), inoculation using syringes and needles (Macy et al. 1972) and with the use of pressurizeable tubes or serum bottles (Balch and Wolfe 1976). The improved Hungate cultivation technique became a mainstream method for the cultivation of anaerobes. To cultivate anaerobes on solid media, Petri dishes filled with solidified agar (agar medium plates) have to be prepared. Incubation of agar plates has to be carried out in anoxic atmosphere or in an anaerobic jar (Fildes and McIntosh 1921). Plating has to be executed in a glove box to guarantee an anaerobic atmosphere. Before transferring the plates into the glove box or tent, the pre-chamber has to be flushed with molecular nitrogen or CO_2_/H_2_-containing test gases, to maintain the anoxic atmosphere in the tent.

### Cultivation vessels and approaches in anaerobic microbiology and biotechnology

Depending on the purpose of cultivating anaerobes to either studying their physiological variables or improving bioprocess parameters for industrial reasons, different cultivation vessels and cultivation conditions can be used. The following section briefly introduce the necessary background information.

#### Cultivation of microorganisms in closed batch systems

**Fig. 4:**
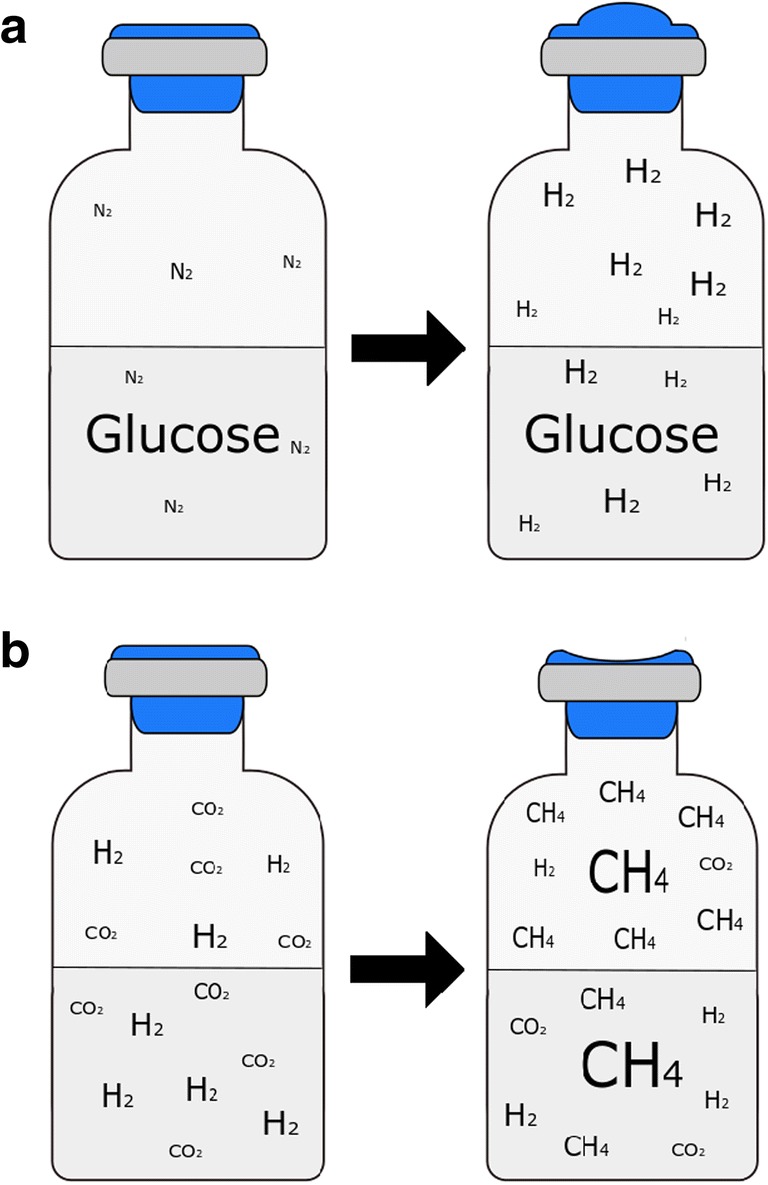
Anaerobic closed batch cultivation set-up (serum bottle) supplemented with (**a**) a liquid substrate, glucose, and (**b**) a gaseous substrate, H_2_/CO_2_. **a** Cultivation of a H_2_-producing microorganism: H_2_ production from glucose leads to a pressure increase in the serum bottle. **b** Cultivation of a methanogenic archaeon: closed batch cultivation with discontinuous H_2_/CO_2_ gassing. The conversion of H_2_/CO_2_ to CH_4_ leads to a pressure drop in the cultivation device due to the following stochiometric formula (4H_2_ + CO_2_ → CH_4_ + 2H_2_O)

To cultivate anaerobes in liquid media, pressurized vessels are filled with sterilized media and the headspace is modified according to the need of the microorganisms (Miller and Wolin 1974; Balch et al. 1979). This set-up is referred as closed batch system (Rittmann and Herwig 2012; Rittmann et al. 2015a, b; Taubner and Rittmann 2016) and it is used to cultivate anaerobic microorganisms in tightly sealed pressure-resistant and crimped sealed glass serum bottles (Fig. 4) (Balch et al. 1979; Taubner and Rittmann 2016). After anaerobization (gas phase exchange with or without boiling the medium), serum bottles can be autoclaved. Before inoculation, an O_2_ scavenging agent such as those shown in Table 1 can be added to remove the residual O_2_ in order to establish a specific ORP. The inoculation process has to be performed with a fixed volume of a defined pre-culture under anaerobic conditions followed by an incubation with or without agitation at the desired cultivation temperature.

If gas producing organisms are cultivated in a closed batch system, excess pressure has to be removed in regular intervals (Fig. 4a). The cultivation of gas-consuming microbes implies the necessity of re-pressurization. The cultivation of gas-converting methanogens is associated with regular supply of, e.g., H_2_/CO_2_. Respectively to the molar stoichiometric gas reduction to CH_4_, under-pressure within the sealed serum bottles could occur (Rittmann et al. 2015a, b; Taubner and Rittmann 2016), see Fig. 4b. As a result of non-continuous gas feeding, the culture could experience non-balanced growth. Nevertheless, closed batch system is a standard anaerobic mircobiological cultivation technique, which allows (1) a fast screening of strains, to determine optimal physical parameters, to investigate the physiological state of the organism, to grow pre-cultures for inoculation, and (2) early development of media composition for further bioreactor studies (Rittmann and Herwig 2012; Rittmann et al. 2015a, b).

#### Cultivation of microorganisms in bioreactors

**Fig. 5:**
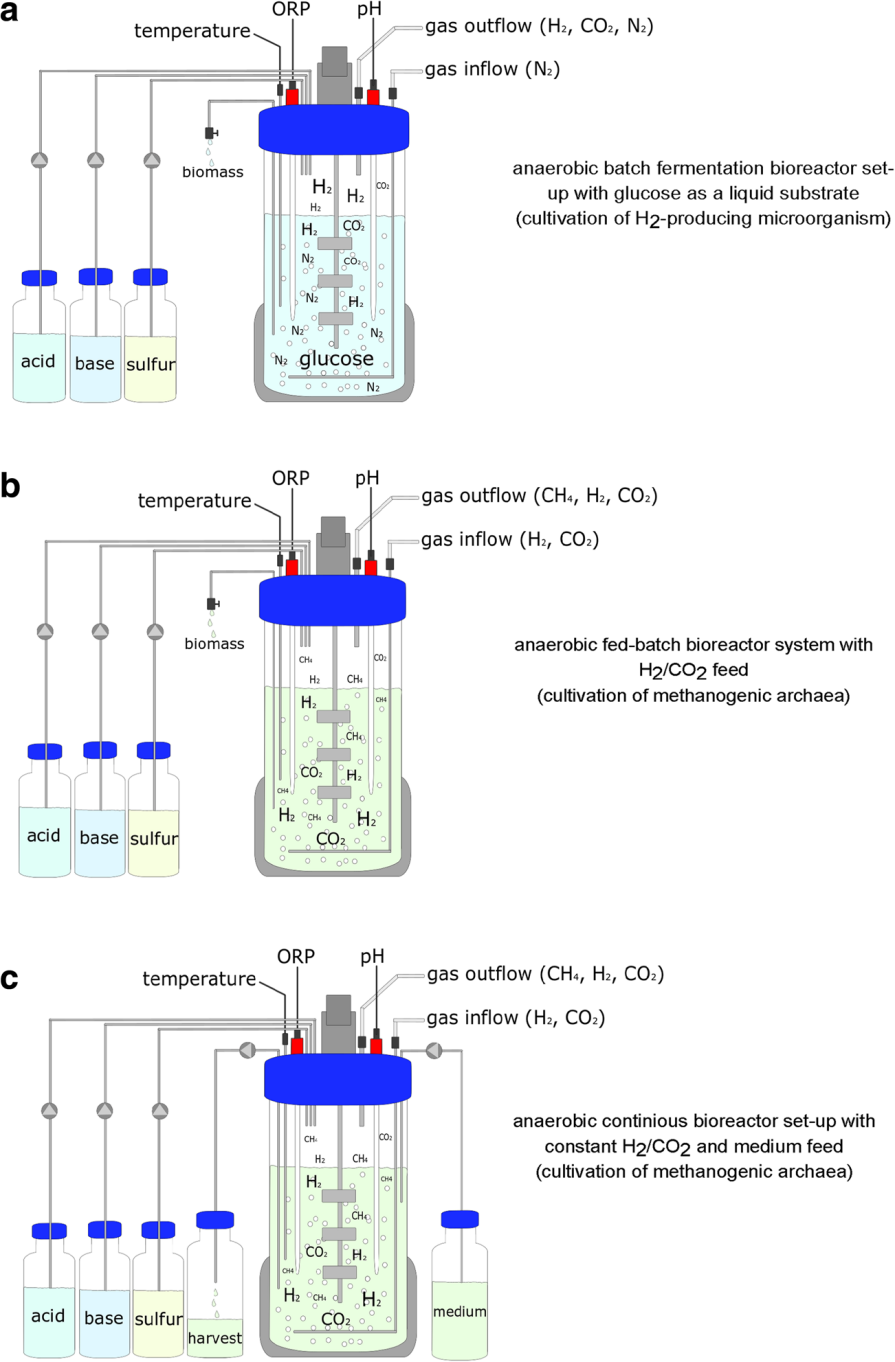
Anaerobic bioreactor systems: **a** batch cultivation, **b** fed-batch cultivation, **c** continuous cultivation. The cultivation temperature is being measured through a temperature probe, which provides information for the heating/cooling control unit (gray covering) of the bioreactor. Liquid feedstocks (acid, base, and sulfur) are pumped into the cultivation vessel. Acid and base are used to control the pH, which is measured by a pH probe. Sulfur/reducing agent (RA) is fed to provide a sufficient sulfur supply and maintain anaerobic conditions during cultivation, which can be tracked via an ORP probe. ORP probes are usually calibrated in an oxic environment via standards. The ORP values can be cross-checked in an anaerobic environment by using the aforementioned redox dyes. Gas is constantly fed during the cultivation (gas inflow) to keep the bioreactor anaerobic (**a**) or to provide substrates (**b, c**). In a continuous bioreactor set-up (**c**), not only gas is fed but also fresh medium is pumped into the system constantly. To keep the bioreactor volume constant, biomass and cultivation broth have to be harvested. Product gas is channeled off by the out gas flow device

Bioreactors are used to cultivate anaerobes in many microbiological research fields, especially in those which are related to industrial microbiology. The most common cultivation approach for microbes in bioreactors are batch, fed-batch, and continuous operation (Fig. 5), and variants thereof, such as semi-continuous cultivation (Macfarlane et al. 1998; Godoy and Meschy 2001; Takeno et al. 2001). In batch cultivation systems (see Fig. 5a), all necessary medium components and inoculum are added at the beginning of the cultivation and no additional feeds are supplemented in the process (Lim and Shin 2013). The biomass concentration will increase exponentially and substrate concentrations decrease exponentially during the cultivation resulting in a (substrate)-limited growth condition when leaving the exponential growth phase. During the cultivation, parameters like pH, temperature, dissolved O_2_ concentration, ORP, and antifoam agents can be applied and eventually controlled. The process can be examined or optimized by changing medium composition, pH, temperature, and other environmental or biotechnological relevant parameters (Lim and Shin 2013).

In comparison to batch cultivation, fed-batch cultivation (Fig. 5b) is mainly used to maintain exponential or linear growth and/or product formation of the microorganism of choice for a longer period of time. Prolonged balanced growth of the microorganisms can only be achieved if a continuous supply of substrates is maintained in a controlled operational procedure. Depending on the microbe and the intended growth conditions, the limiting substrate(s) can either be solid(s), liquid(s), or gases. In general, maximum working volume is defined for bioreactor operations which determines the maximum feed that can be added in a fed-batch operation or the maximum volume at which a chemostat process has to be controlled. Hence, the key aspect is to control the cultivation volume below that maximum working volume. This can be done by, e.g., measuring the reactor volume or eventually by applying process balance concept in order to predict the volume variation.

Continuous cultivation includes a continuous inflow of media and continuous outflow of suspension (Chmiel 2011), see Fig. 5c. Other continuous cultivation systems employ a cell retention system when the maximum division rate of a given strain in given conditions is found to be below the needed dilution rate. If a continuous culture cell retention system is in operation, also a steady state can be reached through setting up a feed, bleed, and cell recycling system (Okabe et al. 1994; Richter and Nottelmann 2004; Deschênes et al. 2006). When a population of cells is grown in steady-state mode, which means growth at a fixed average *μ*, even though not all continuous cultures can be operated or are able to reach a steady state. In a physiological steady-state experiment, the concentrations of biomass, substrate, and products reach an equilibrium and are “independent of time”. A physiological steady state is generally expected after 5 volume exchanges (Chatzifragkou et al. 2010). Eventually, all of the above mentioned cultivation conditions can be applied in dynamic experiments to investigate physiological variables in order to optimize a bioprocess performance (Spadiut et al. 2013).

### Sampling methods, issues, and challenges

Obtaining representative samples of biomass in an anaerobic bioreactor system is required to correctly quantify a cultivation system. A correct sampling procedure needs to respect two important criteria (Smith 2001):

- The sample need to exhibit an homogeneous distribution of the quantified particles in order to be representative for the entire system undergoing quantification.
- The sample is preserved during and after the sampling procedure until quantification is performed.

Since not every anaerobic cultivation and subsequent sampling can be performed in an anaerobic chamber, the working procedure has to be adapted to avoid a possible contamination with O_2_. For sampling from closed batch, batch, fed-batch, and from continuous culture systems, which are operated under standard atmosphere, gassing cannulas attached to an O_2_-free gas source are commonly used to maintain anaerobiosis in the culture vessel as well as in the sample (Bacic and Smith 2008). If overpressure is expected in the culture vessel (tubes or serum bottles), pressure has to be released with a sterile syringe in advance to prevent the vessel from reaching a pressure above the tolerated values. The same volume of gas that was withdrawn during sampling of the O_2_-free gas has to be re-injected into the vial to prevent vacuum to be build up. The vial may be turned upside down to fill the syringe with the required amount of liquid sample and afterwards removed carefully (Miller and Wolin 1974). Withdrawing a representative sample from a pressurized bioreactor could also lead to problems due to potentially occurring cell lysis caused by the sudden pressure drop. It was found that *Methanocaldococcus jannaschii* cultures exhibited cell lysis when the culture was decompressed (Park and Clark 2002), whereas the measurement using the Bio-Rad microassay technique yielded an increase of protein concentration independent of decompression time. However, the optical density was strongly affected by decompression (Miller et al. 1988; Park and Clark 2002). However, studies on the effect of hydrostatic pressure to *E. coli* 15 and further liquid sampling by decompression and compression within 5 s did not result in lysis or decreasing cell growth after sampling (Yayanos and Pollard 1969; Yayanos 1975). These investigations lead to the assumption that the described sampling procedures have different effects depending on the investigated strain. Sampling procedures without decompression of the bioreactor system have not been developed yet, but they would improve quantification in high-pressure bioprocesses.

If the biomass quantification is not of interest, withdrawal of the liquid suspension for quantification purposes can be done by different means. Sampling systems like filtration probes or dialysis-filtration-sampling probes are solutions for obtaining representative and real-time samples of a process. These probes are constructed as a dip tube, which continuously extracts biomass from the culture broth. Further on, low molecular weight substances can diffuse through a membrane into a buffer stream, where the analyte becomes diluted, but prevents a change in volume as well as from contamination. Pressure differences between the reactor and dialysis system should be avoided (Chmiel 2011). Independent of the used cultivation conditions or vessels, the sampling procedure should avoid perturbing the process operation. Thus, choosing the right procedure and correct sampling approach should improve the quantification outcomes.

## Analytical approaches for quantification of biomass

Depending on the constraint of a selected method for quantification, it will be possible to perform an analysis of a target compound by using offline, at-line, and/or online sampling approaches. Offline biomass quantification approaches are well applied and already approved in biotechnological processes, although the workload to get to the desired result is enhanced compared to at-line or online quantification approaches. Further, offline strategies entail the risks of contaminating the cultivation vessel or the sample itself. At-line measurements have one major advantage over traditional offline techniques; the sampling is performed automatically in prescribed intervals. Respectively, at-line biomass quantification is close to real-time analysis. If the installations of online quantification devices are not feasible due to technical issues, available space or financial reasons at-line measurement applications could be applied. Besides that, online biomass quantification approaches are preferred over offline and at-line strategies. At-line biomass quantification approaches reduce the amount of work involved in sampling, even though there are some essential points which have to be considered as they are (1) the transfer of the sample to the measurement device, (2) conditions during the transfer, (3) the homogeneity of the sample, (4) the representativeness of the sample for the whole cultivation, and (5) the recycling of the sample (bypass loop) or the discharge of the sample after measuring.

Online biomass quantification approaches have plenty of advantages over offline and at-line strategies. Sampling and the transfer to the measurement device is being circumvented since the measurement is performed directly in the cultivation vessel or bioreactor. Therefore, no time delay between sampling and measurement in addition of the analysis time itself has to be taken into account for data analysis (Vojinović et al. 2006). The direct measurement in the cultivation vessel can potentially reduce also the risk of contaminating the bioreactor and the possible intoxication of the operator by toxics compounds or pathogenic microorganism (Höpfner et al. 2010). But for that, CIP (clean in place) and SIP (sterilization in place) strategies has to be validated with the compatibility of the used measuring device. Besides that, the bioreactor volume needs to be sufficient for a given measuring device and the recalibration of the equipment has to be considered. In continuous processes, analytic probes/equipment can also be placed in a bypass loop in order to facilitate recalibration and exchange in case of failure, but the representativity of the sample needs to be assessed even though data interpretation and data validation can be challenging. Real-time monitoring gives direct insight into a bioprocess and further information about specific productivities and total yield (Sandnes et al. 2006). Online sensors stand out with their flexible and detailed data processing, while the analyte remains unaffected. Depending on the required information, whether gaining additional data about the concentration of medium components beside the biomass concentration or the viability of a culture, different sensors can be individually introduced into a bioreactor system.

### Offline biomass quantification approaches

Biomass quantification can be performed by applying several different methods, all possessing some advantages or disadvantages. Especially when working with offline biomass quantification approaches, washing and purification steps have sometimes to be encountered to be able to quantify the amount of produced biomass.

In this review, we will categorize offline techniques into five different subsections:

1. Direct cell counting
2. Colony counting
3. Most Probable Number (MPN)
4. Biomass measurement
5. Light scattering

#### Direct cell counting

Direct cell counting sums up all methods based on the enumeration of detectable cells within a liquid medium and consists of:

- Microscopic enumeration
- Electronic enumeration
- Fluorescence Activated Cell Sorting (FACS)

##### Microscopic enumeration

Microscopic enumeration is another term for cell counting. Counting of single cells can be performed by using different approaches. One of the most common approaches is microscopic enumeration that can either rely on using a membrane filter sampling technique (Brock 1983), followed by a cell or nucleus staining procedure (Koch 2007), or by using a counting chamber. Counting chambers are a well applied microbiological tool to directly count cells. Depending on the microorganism, different counting chambers and microscope settings can be applied. For counting bacteria, commonly counting chambers with counting chambers depth of 0.02 mm are used, whereas for counting larger microbes like yeast or algae, a counting chamber depth of 0.1 mm should be preferably applied (Bast 2001b). The two main disadvantages of direct cell counting are the reproducible filling of the counting chamber and the adherence of cells on the glassware surfaces and pipette tip. The market offers a great variety of counting chambers which usually differs in the applicable volume, design of the counting grids and compatibility with different objectives. Besides that, every counting chamber is calibrated for specific objective types. For instance, Neubauer counting cambers are suited for high-dry objectives (Talking et al. 2014), whereas Hawksley counting chambers can be used under oil-immersion objectives (Koch 2007) which are, e.g. more suited for counting small-sized cells. However, without using a cell staining method, distinction between viable, dormant, and dead cells is not possible (Talking et al. 2014). The use of a counting chamber is eased when applying autofluorescent strains. This approach enhances the visibility of cells by excitation of cellular compounds at a specific wavelength, e.g. the UV-inducible blue-green autofluorescence of microorganisms. Many H_2_-utilizing methanogens can be counted by exposing them to an UV light, subsequently strain originated autofluorescence is induced by special cofactors. Coenzyme F_420_ absorbs light at a wavelength of 420 nm and emits blue-green light, which can be detected by a fluorescence microscope (Solera et al. 2001; Kumar et al. 2011). Deazaflavin F_420_ functions as an essential coenzyme within the methanogenesis pathway. The reduced form of F_420_ (F_420_H_2_) functions as an electron donor for methylenetetrahydromethanopterin dehydrogenase (Mtd), cysteine-containing F_420_-reducing hydrogenase (Frc), and for selenocysteine-containing F_420_-reducing hydrogenase (Fru) (Hendrickson and Leigh 2008). However, due to their low coenzyme F_420_ content, counting of acetoclasic methanogens is rather difficult (Kamagata and Mikami 1991; Solera et al. 2001). Another aspect that needs to be considered when applying these enumeration methods is the aggregation state of biomass. For example, *Methanosarcina* spp. may form aggregates under certain environmental conditions, which complicates counting of single cells by autofluorescence cell enumeration (Solera et al. 2001). Counting autofluorescent methanogens during cultivation in bioreactors is frequently used (Ahn et al. 2000; Solera et al. 2001). The autofluorescence of methanogens could also be used to distinguish methanogens in co-cultures from other microbes which do not express the coenzyme F_420_.

##### Electronic enumeration

Electronic enumeration of cells is another approach for determining the cell number. The Coulter counter is routinely used in clinical hematology and for the enumeration of non-filamentous yeast and protozoa. However, this technique is hard to apply to bacteria and other microbes with similar morphological characteristics, like small cell size and elongated shape (Kubitschek 1969).

##### FACS

FACS allows the measurement of scattered light and fluorescence emissions produced by illuminated single cells that are passing through a capillary that is intersected by a laser beam. Once a cell passes through a beam of light a signal is produced. The scattered light and fluorescence emissions of each cell are collected by detectors and are further processed in silico. The in silico process allows the distribution a population with respect to different parameters measured by a given equipment. Forward scattered light, collected in the same direction as the incident light, is related to cell size. Collected side scattered light (angle of 90°) provides information of cell surface properties and internal structure of the cell. Information concerning the cell is obtained by staining the sample with different fluorochromes (Álvarez-Barrientos et al. 2000; Lehtinen 2007). Most FACS have been limited to aerobic microbial systems due to the oxygenated atmosphere of the sort stream and the cell deposition. To test the viability and sort cells, a BD (BD Bioscience) Influx cell sorter was modified for anaerobic working conditions by purging O_2_ from the sort stream and cell deposition areas (Thompson et al. 2015). This group showed the utility of this device for separating anaerobic target populations from co-cultures, however the method can easily be expanded to the isolation, genotyping, and cultivation of anaerobic microorganisms sorted from complex natural communities.

#### Colony counting

The amount of viable microorganisms can be elucidated by colony counting (Hungate 1969). This technique can be performed by

1. Spreading the diluted sample over a solid agar (spread plate method)
2. Pipetting the culture into a sterile Petri plate and mixing it with molten agar medium (pour plate method) (Postgate 1969)
3. Pipetting a sample into a small amount of molten but cool agar medium (bearable temperature for the microbe), followed by pouring the mixture onto a sterile agar plate, allowing it to harden (thin layer plates)
4. Using the thin layer technique, but adding another agar layer on top of it (layered plates)
5. Filtering the diluted sample with a pre-sterilized filter and placing it onto the sterile agar medium plate (membrane filter method).

In anaerobic microbiology, all these techniques are utilized, but compared to aerobic conditions they require some additional precautions. For solid media, the execution of the colony counting methods (b.1–b.5) has to be carried out under anaerobic conditions. This can be realized by making the media anoxic, counting in a glove box or by using an anoxic chamber for inoculation. In most cases, the samples have to be diluted before plating to obtain an adequate quantity of colony-forming units (CFUs). This number generally lies between 30 and 300 colonies per plate (Sutton 2011). Dilution of samples is a sensitive step since it needs to be compatible with the physiological requirements of the microbe in respect to pH and osmolality (Koch 2007). After preparing and incubating the agar plates, CFUs may be determined by using an appropriate period. However, CFUs mostly consists out of more than one initial starting cell, which must be considered as well (Li et al. 1996; Lehtinen 2007; Madigan et al. 2012). The techniques in this section can only detect viable and culturable microorganisms. Dormant, non-culturable microbes, and microorganisms with very low *μ* are not detected with the previously described methods (Barer and Harwood 1999; Oliver 2005).

##### MPN

The concentration of viable cells in culture can be estimated by applying the MPN method. The amount of proliferating microbes is determined with MPN by the amount of dilutions, where growth is observable (Kott 1966). This method is based on statistics. MPN has already been applied for anaerobes, especially for estimating the methanogenic population in an anaerobic thermophilic digester and a mesophilic soil sample (Wagner et al. 2012).

##### Biomass measurement methods

Sometimes it might be preferred to assess the cell mass instead of the real number of cells. Biomass can be measured by determining wet weight or dry weight of a culture sample (Tisa et al. 1982; Guerrero et al. 1985). Cell dry weight is determined by drying pelleted biomass for a defined period of time with approximately 105 °C in glass eprouvettes (Koch 2007), subsequently followed by cooling in a desiccator and weighing. As dry mass corresponds to 10–20% (*m*/*v*) of the wet mass (Madigan et al. 2012), also the wet mass can be determined. Wet mass can simply be obtained after centrifugation of the sample and removing of the supernatant (Tisa et al. 1982; Troller 1989). After this process, a packed cell pellet remains, which should be weighed to determine the wet mass (Tisa et al. 1982). The quantification of biomass dry or wet weight can be correlated to other biomass quantification approaches such as spectrophotometry. Furthermore, for improving bioprocess quantification, the elementary composition of biomass can be determined (Mauerhofer et al. 2018) to balance growth stoichiometry on an elemental molar basis.

##### Light scattering

Light scattering methods are mostly used to monitor the growth of pure cultures (Günther and Bergter 1971). However, methods based on light scattering give mainly information corresponding molecular content/ dry weight and not about the number of cells (Koch 1970). The cell biomass can be estimated through the turbidity of a culture, which is measured with a photometer (fix wavelength) or spectrophotometer (whole wavelength spectrum). The principle of this measurement is based on the absorption of light by cells in the suspension at a certain wavelength; but only unscattered light is detected. The amount of cells in the light path decreases the intensity of the incident light beam and gives an indirect correlation of the amount of biomass in the sample. The method of turbidity measurements is better known as determination of the optical density (OD) (Koch 1970; Koch 2007). The more cells are in the suspension the more light is scattered or absorbed and less light can be detected (Madigan et al. 2012). This correlation is described by the Beer–Lambert law, see Eq. 2 (Bast 2001b). The Beer–Lambert law is empirically valid only for OD values < 0.5 (Locher et al. 1992) because of light scattering effects increase with higher cell density. The incoming light beam gets initially scattered by the cells (primary scattered light). If the amount of cells is too high, the possibility for scattering already scattered light (secondary scattered light) is increased, which results in measuring lower OD values than the real extinction value. However, with the preparation of standard curves and appropriate dilution series measuring up to higher OD values is possible (Bast 2001b). A relation between the cell dry weight and the absorbance was found to be directly proportional and shows a linear correlation (Koch 1961).

2 $$ {\varPhi}_{ex}={\varPhi}_{in}\cdot {e}^{-{s}_n\cdot c\cdot d} $$

In Equation 2 *Φ*_*ex*_ (W m^−2^) is the intensity of the incident light, *Φ*_*in*_ (W m^−2^) is the intensity of outgoing light, s(m^2^ mol^−1^) is described as the scattering coefficient, c(mol L^−1^) is the concentration of the cell suspension, and d(m) is the layer thickness. Offline turbidity measurements are being executed by an external photometer. Therefore, a small amount of biomass (up to 1 mL) has to be harvested, further transferred into a dedicated cuvette, and measured at a proper wavelength. Microplate systems in contrary to cuvette spectrophotometers allow measurements even with 100 μL of harvested suspension (Stieber et al. 1994; Turcotte et al. 2004). Investigations on different spectrophotometers showed a high dependency in the OD measurements in respect to geometry and the optical design resulting in different OD values for the same cell suspension. This has to be taken into account when performing measurements with different systems. OD measurements can only be compared when measuring with one specific spectrophotometer. Then OD-based biomass quantification can be correlated to other offline biomass quantification methods. However, the correlation of biomass concentration to light scattering must be individually determined for each organism and growth media. Moreover, the correlation is only valid in a specific range as discussed above.

When performing OD measurements, medium characteristics have to be taken in account, since quantification of microbes within the medium could be affected. Some medium components could impede the quantification of microbes via light scattering, especially when working with dark samples from a digester or manure plant. To overcome darkness, samples including blank could be diluted, which have to be considered later when elucidating the amount of cells. If a dilution is not realizable, due to immense microbial biomass loss, other biomass determination techniques have to be investigated.

### At-line biomass measurement

At-line measurements represent an improvement over traditional offline methods and are close to real-time analysis; of course the ideal approach is monitoring online, preferably in situ. However, the installation of online measuring devices is not feasible at each bioprocess condition.

Commonly anaerobic digestion plants are regulated based on at-line or offline analytical results (Madsen et al. 2011). By applying an at-line attenuated total reflectance-mid-infrared (ATR-MIR) spectroscopy, ammonium, glucose, methyl oleate, and biomass were investigated in a complex antibiotic fermentation process using *Streptomyces clavuligerus* (Roychoudhury et al. 2006). At-line information gathered from flow cytometry can also be used to change the biofuel production control strategy to enhance the process yield (da Silva et al. 2012). In principle, almost every measuring deceive can be installed at-line.

### Online biomass measurement

The most common in situ measurement devices (Vojinović et al. 2006; Kiviharju et al. 2008; Höpfner et al. 2010) are as follows:

1. Optical sensors
2. Fluorescence optical sensors
3. Other spectroscopic sensors

#### Optical sensors

Measurements of biomass with optical sensors are either based on transmission or backscattering. Probes based on the backscattering principle do not show any limitation in case of increasing biomass concentration compared to transmission probes. Visible optical sensors can produce erroneous responses caused by cell morphology, or interfering gas bubbles (Ulber et al. 2003; Vojinović et al. 2006). Other suspended effects, and the necessity for cleaning of optical sensors are common problems of these probes (Locher et al. 1992). Individual calibration for optical sensors is recommended since the signals depend strongly on the cell morphology. Measurements of cell dry weight and optical online methods showed different correlations according to the investigated strains (Ude et al. 2014).

#### Fluorescence optical sensors

Fluorescence optical sensors can be employed to measure lifetime fluorescence emitted by microbes in a culture. When applying this method, only viable cells in the population can be detected. In active and living cells NAD(P)H plays an important role for the electron transfer from electron donor to electron acceptor. The signal and amount of NAD(P)H in a biological system was found to correlate with the biomass concentration (Coppella and Rao 1990; Farabegoli et al. 2003). This technique is limited respectively to inferences from medium compounds that emit or absorb between 360 and 450 nm. Therefore, only well-defined medium compositions can be used when applying optical sensors (Marose et al. 1999). Possible interferences by several fluorophores (e.g. FAD, NAD, NADH) can be circumvented with 2D absorption/emission fluorescent spectra measurements or multi-wavelength fluorometry (Morel et al. 2004; Vojinović et al. 2006; Kiviharju et al. 2008). The robustness and the capability of measuring intracellular effects as well as their rapidity in measuring of fluorescent samples are the main advantages of these systems (Locher et al. 1992).

#### Other spectroscopic sensors

##### Infrared spectroscopy

Spectroscopic sensors are commonly used to detect infrared light within a range of 0.74–1.00 nm (Landgrebe et al. 2010). Infrared spectroscopy is an analytical technique which is used to analyze a wide variety of organic compounds, substrates, products, metabolites, and biomass. This method is based on molecular vibrations of organic compounds, which have spectral signatures that belong to the infrared domain (Landgrebe et al. 2010). The infrared light is subdivided into three regions: far infrared (FIR), mid-infrared (MIR) and near-infrared (NIR) region. To monitor bioprocesses, two spectroscopic sensor types are available, MIR and NIR probes (Olsson and Nielsen 1997; Landgrebe et al. 2010). Microbial growth can be either measured via light absorption (turbidity) or light scattering (nephelometry) in the visible and NIR ranges (Marose et al. 1999). NIR shows the best correlation between wavelength and biomass at 2300 nm. The majority of media do not absorb light in this NIR region (2300 nm) (Olsson and Nielsen 1997; Marose et al. 1999).

##### Electrochemical impedance spectroscopy

Low frequency electrochemical impedance spectroscopy (EIS) can be used as an online process tool to monitor viable cell concentrations during cultivations. Via EIS, the relative permittivity between two electrodes affected by cells with an integer cell membrane is detected. This signal is in turn correlated to cell dry weight measurement of the organism of interest. Thus, estimation of viable cell concentration can be conducted. The proposed technique has a high dynamic range from low to high cell densities beyond 40 g/L^-1^ cell dry weight with low background interferences (Slouka et al. 2016).

### Modeling of growth kinetics

Modeling is a powerful tool to get insight into a biological bioprocess. Modeling concepts are mentioned below:

1. State estimation
2. Estimation of volumetric mass bio-density
3. AMD1 model

#### State estimation

Real-time monitoring of physiological characteristics such as biomass, product, substrate, and precursor concentrations during cultivation is of great importance during biotechnological processes. Particle filter algorithm could be applied for estimating these difficult-to-measure process states. The particle filter represents a new algorithmic framework, combining several already existing methods and techniques (online and offline) for state estimation (Kager et al. 2018).

#### Estimation of volumetric mass bio-density

The biological biomass density (biomass/bio-volume) referred as bio-density is a physiological variable that can be estimated by using dielectric spectroscopy and a soft sensor based on first principle elemental balances. The combination of both signals allows a real-time estimation of the bio-density during cultivation. Dielectric spectroscopy measures the permittivity of the fermentation broth in dual frequency mode, a high frequency accounting for non-cellular background and a low frequency accounting for the permittivity attributed to living cells. Dielectric spectroscopy estimates the biomass via correlating the permittivity signal, which reflects the encapsulated volume fraction of cells. Soft sensors are software algorithms that calculate non-measured process parameters from readily available process signals. Accurate estimation of the biomass concentration via elemental balancing can be performed. The application of this sensor allows a real-time calculation of specific rates and yield coefficients, which provides insight to physiological changes. The combination of both signals, dielectric spectroscopy and soft sensor, provides a possibility to estimate the volumetric mass (Ehgartner et al. 2014, 2017).

#### ADM1 model

The anaerobic digestion model No. 1 (ADM1) reflects the major processes steps during digestion and product formation, conversion of complex organic substrates into CH_4_ and CO_2_ and inert by-products (Batstone et al. 2002; Jimenez et al. 2015). The kinetic equations consider microbial growth and biomass decay. Therefore, the model incorporates seven microbial trophic groups. Growth of these groups is related to degradation rates of organic matter and is described by Monod-like dependencies. Also, inhibitive effects of pH, H_2_, ammonium, and fatty acids are considered by equations. The model includes the degradation of complex solids into carbohydrates, proteins, and fats, which get further hydrolyzed to sugars, amino acids, and VFAs. Carbohydrates and proteins are fermented to VFA (acidogenesis) and H_2_. Fatty acids are converted into acetate and H_2_. CH_4_ is produced by acetoclastic and autotrophic, hydrogenotrophic methanogenesis. The physicochemical equations describe ion association and dissociation, and gas–liquid transfer during the digestion process. This differential and algebraic equation set enables the determination of 26 dynamic state concentration variables, and 8 implicit algebraic variables per bioreactor vessel or element. For monitoring of the process, there are further 32 dynamic concentration state variables provided, based on differential equations (Batstone et al. 2002; Jimenez et al. 2015). The ADM1d model is an extension of the ADM1 model and describes biomass distribution within a one-compartment model (Mu et al. 2008).

## Discussion—analytical approaches for quantification of biomass

Microbial growth during a cultivation should to be monitored. Biomass quantification can be targeted by using offline, at-line, and/or online approaches. The usage of offline direct cell counting, including microscopic enumeration, electronic enumeration, and FACS implies the possibility to count microbes in liquid media, although only a representative sample volume is used to determine the number of cells. Direct cell counting techniques facilitate the determination of microbes in liquid media without the requirement of turbidity compared to light scattering technique. Under ideal conditions, medium characteristics should not affect the quantification of microbes within the medium, although medium components could impede the quantification of microbes, e.g. digester or manure samples, because they are mostly dark and of high viscosity. To overcome darkness or viscosity, samples can be diluted, which have to be considered later when elucidating the amount of cells. If a dilution is not applicable, indirect biomass determination techniques that are based on substrate consumption, product formation, or biomass viability investigations can be employed. Moreover, complex media compounds or polymeric substances can also impede proper quantification (Reischl et al. 2018b). Microscopic enumeration is more cost-efficient than electronic enumeration and FACS, although susceptibility of errors is increased. Determination of growth through most probable number technique is easy to perform. Although it has some disadvantages over direct cell counting, as they are, contaminations are not detectable, cells are not counted, and only the amount of viable cell is being estimated. Growth determination on solid media could be performed via colony counting. Colony counting does not allow the elucidation of the actual cell number. Instead, growth is indicated by colonies which have to be counted. Instead of counting colonies, wet and dry weight determination can be elucidated. This method gives only an insight in weight increase or decrease and no accurate determination of cell number. Additionally, OD measurements should be performed. When performing OD measurements, the medium absorption have to be considered too. Depending on the purpose and the available budget, different applications are possible.

Online biomass measurements provide the possibility to monitor microbial growth in real time. Optical sensors detect cells directly, thus signals generated by optical sensors are strongly dependent on the cell morphology, which could also produce erroneous responses. Whereas fluorescence optical sensors measure lifetime fluorescence emitted by microbes, here only viable microbes can be detected (Coppella and Rao 1990; Farabegoli et al. 2003). Low-frequency electrochemical impedance spectroscopy (EIS) can be used as an online process tool to monitor viable cell concentrations during cultivations. Microbial growth could also be quantified by using a modeling strategy to estimate biomass increase during the cultivation process. This strategy is cost-efficient since not every parameter (e.g. biomass, substrate, and product) has to be detected by a single device. As space for measurement devices in cultivation vessels is limited, this modeling strategy could improve the monitoring of the cultivation process. The ADM1 model has been specifically developed for modeling of anaerobic digestion bioprocesses. This modeling strategy is well applied and further advancements have been intended.

## Quantification of live and dead biomass

“What is life?” Life is a biochemical process or an energy flux in a biological system. The trial to answer this question leads to the reverse questioning “What is death?” The philosophical distinction between life and dead is problematic (Davey 2011), which is also true in microbiology. According to Martin et al. (2014), the core of the living process of all organisms is based on energy-releasing chemical reactions or metabolic energy (adenosine triphosphate (ATP)). Therefore, life could be seen as a generation of metabolic energy within a defined compartment, envelope, or membrane. On the other hand, death could be interpreted as lacking ATP production in the organism. Generally, the determination of microbial viability under certain conditions is essential to be able to control and monitor their productivity. The monitoring of viability has a great importance in many fields of microbiology and even beyond such as in food production (Ercolini 2004), health care sector (hospital) (Galvin et al. 2012), ground water sustenance (Clinton Ezekwe and Nwabuko Chima 2013), production of pharmaceuticals (Jimenez 2004), and biological product generation (Gaylarde et al. 1999). To determine the physiological status of an anaerobic population, knowledge of the amount of alive and dead cells in the population is relevant. Therefore, some methods have been implemented to study the viability of anaerobes. Those methods can be divided into the following groups:

1. Staining and quantification of biomass by microscope and FACS
2. Viable biomass quantification by molecular methods
3. Quantification of viable biomass by using physiochemical parameters

### Staining and quantification of biomass by microscope and FACS

There are several staining methods available to investigate the viability status of microbes:

- LIVE/DEAD BacLight® bacterial viability kit
- LDS-FISH
- BONCAT
- BONCAT-FISH
- BONCAT-FACS
- Microautoradiography (MAR)

The detection of live and of dead cells can be either performed by microscopy or by using cell sorting. Both detection techniques are discussed in the sections below.

#### Live/dead

The BacLight^®^ bacterial viability kit staining can be used for the application of microscopy and FACS. This kit was initially developed to investigate the viability of bacteria. The usage for archaea has already been confirmed by some research groups, which are mentioned below. LIVE/DEAD BacLight^®^ bacterial viability kit is offered for instance by the company MOLECULAR PROBES EUROPE BV Leiden (Netherlands, www.probes.com). The two-color fluorescence assay can be used for the distinction between live and dead microbes. It provides a mixture of the green (SYTO 9) and red (propidium iodide (PI)) fluorescent nucleic acid stains. Both stains differ in their spectral characteristics and in their ability to penetrate viable cells. When SYTO 9 stain is used separately, all microbes with intact and damaged membranes get labeled. In contrast, propidium iodide penetrates only microbes with damaged membranes. Subsequently, a reduction of SYTO 9 stain fluorescence is induced. Through appropriate mixture of both stains, microbes with intact cell membranes stain fluorescent green, while microbes with defective membranes stain fluorescent red. The excitation/emission maxima for these dyes are about 480–500 nm for SYTO 9 stain and 490–635 nm for PI. The kit is well suited for fluorescence microscopy or for the application in quantitative analysis with a fluorometer, fluorescence microplate reader, flow cytometer, or other instrumentation. The LIVE/DEAD BacLight kit^®^ was initially developed for investigations of vital and dead bacteria, but it is already used in a broad range of application in Microbiology. The intolerance of haloarchaea species, except halococci, to distilled water (Garrity et al. 2001) was used for investigating the reliability of the BacLight kit^®^ to detect extremophilic archaea (Leuko et al. 2004). *Halobacterium* sp. strain NRC-1 was chosen as a reference strain to detect dead haloarchaea (reduction of SYTO 9 by propidium iodide “red fluorescence”) as it lyses in presence of distilled water easily, and cells of *Halococcus dombroskii* H4 were used as reference to detect vital haloarchaea (SYTO 9 “green fluorescence”) (Leuko et al. 2004). Also, the incubation with LIVE/DEAD BacLight kit^®^ reagents SYTO 9 and PI for up to 24 h did not noticeably reduce the growth of the two haloarchaeal species. To summarize, the LIVE/DEAD BacLight kit^®^ could be used to assess the viability of haloarchaea (Leuko et al. 2004). Further, the cultivability is not affected upon usage of the kit up to 24 h. LIVE/DEAD^®^*BacLight™* kit can be used to study the viability of psychrophilic archaea (Moissl et al. 2003). By applying this kit the physiological status of the SM1 euryarchaeal cells at 10 °C in sterilized marsh water (pH 6.5) was evaluated. The staining indicated a cell viability of 90%. The applicability of the LIVE/DEAD^®^*BacLight*™ kit was also tested for methanogenic archaea (*Methanobacterium lacus*, *Methanobacterium movilense*, *Methanosarcina soligelidi*, *Methanosarcina barkeri*) (Heise et al. 2016). The strains were stained before and after isopropanol killing procedure. SYTO 9 stained all archaeal cells, whereas PI only penetrates cells with damaged membranes. After isopropanol killing, both *Methanosarcina* spp. formed defense aggregates of cells and medium components. The cell wall structure of single cell *Methanosarcina* spp. consists of a fairly porous surface layer called S-layer, aggregated cells are encapsulated in a methanochondroitin sheath (Sowers et al. 1993). Possibly, this method is not suitable for aggregated *Methanosarcina* spp. to allow distinguishing between live and dead cells.

#### LDS-FISH

LDS-FISH is another visualization method to differentiate alive and dead cells (Savichtcheva et al. 2005). This method combines fluorescence-based live/dead staining and FISH; it is applicable for microscopy and FACS (Álvarez-Barrientos et al. 2000; Lehtinen 2007). By applying LDS-FISH, the viability and survival ability of fecal *Bacteroides* spp. in environmental waters was tested (Savichtcheva et al. 2005). The authors successfully demonstrated that LDS-FISH method is a powerful tool to monitor the viability of anaerobic fecal *Bacteroides* spp. in drinking water. The combination of both methods, allows the detection of single microbes (FISH) and determining their viability status.

#### BONCAT

BONCAT is used for visualizing transcriptional active cell of either archaeal or bacterial pure cultures inside of complex samples, for instance, biofilms, freshwater, and anoxic sediments. This method is based on in vivo incorporation of the non-canonical amino acid l-azidohomoalanine (AHA). AHA-containing cellular proteins get further fluorescently labeled by azide-alkyne click chemistry (Hatzenpichler et al. 2014).

#### BONCAT-FISH

The advantage of combining BONCAT and FISH (BONCAT-FISH) is based on the specific labeling of transcriptional active cells within complex samples like biofilms. Through this method, newly synthesized proteins can be detected via BONCAT, in combination specific strains can be identified via rRNA-targeted FISH. As a control, 4′,6-diamidino-2-phenylindole (DAPI) can be applied to stain all cells. For quantification of transcriptional active cells, overlay programs can be used (Hatzenpichler et al. 2014).

#### BONCAT-FACS

A novel approach combining BONCAT with fluorescence-activated cell sorting, referred to as BONCAT-FACS, is to separate translationally active cells by tracking the incorporation of synthetic amino acids into newly synthesized proteins from complex samples. By applying this technique the authors were able to directly link the identities of anaerobic CH_4_-oxidizing archaea with their partner bacteria and detect transcriptional active cells (Hatzenpichler et al. 2016).

#### MAR

MAR is a well-applied method in the aquatic and terrestrial microbiology field to measure single-cell activity. This method enables a direct visualization of active cells and their metabolic capabilities without prior enrichment or cultivation (Nielsen and Nielsen 2010). The method is based on a short-term incubation of radioactive-labeled substrate. Those substrates get up-taken by individual cells, which can be visualized by an autoradiographic emulsion. This emulsion is placed on top of the radioactive-labeled organisms and subsequently processed by standard photographic procedures. Excited silver ions will precipitate as metallic silver, resulting in silver grain formation adjacent to or on top of the active microbial cells. Those cells can be visualized under the bright-field or phase-contrast microscope (Nielsen et al. 2003).

### Viable biomass quantification by molecular methods

Before investigating the viability of a microbial population via a molecular based method is performed, DNA and/or has to be extracted. Special medium characteristics or environmental conditions can interfere with the extraction method (Rittmann and Holubar 2014). Those features have to elucidate before starting the extraction or quantification and the applied methods have to be adapted. When applying this technique for a strain that has not been investigated yet with this method, adjustments have to be initially performed. The viability of microbes can also be studied by using molecular based methods, like:

1. PMA-qPCR
2. DNase I/Proteinase K
3. RNA analysis
4. Genomics

#### PMA*-*qPCR

The analysis of viable and dead cells in a population could be investigated by applying quantitative PCR with prior propidium monoazide (PMA) treatment (Heise et al. 2016). PMA is a DNA-intercalating dye. Due to its positive charge, PMA is incapable of penetrating cells with intact cell membranes, but it selectively interfuses membrane-compromised cells. The photo-inducible azide group of PMA can be converted into a highly reactive nitrene radical which binds covalently to free DNA upon exposure to bright light (Nocker et al. 2006). Respectively to the masking nature of PMA toward free DNA, qPCR amplification results only in amplicons from intact cells (Nocker and Camper 2009). It was shown that PMA-qPCR technique is suitable for the differentiation between live and dead methanogens (Heise et al. 2016). Further findings indicate that unscathed membranes of methanogens have a natural barrier for PMA (50–130 μM, < 20 min). Thus, PMA can be used for detecting a lack of membrane integrity. The company Biotium (https://biotium.com) commercially distributes a LED photolysis device (PMA-Lite™ LED Photolysis Device), specifically designed for photoactivation of PMA, ethidium bromide monoazide (EMA), or other similar azido dyes.

#### DNase I/Proteinase K

An alternative method for the discrimination between live and dead cells is the DNase I/Proteinase K treatment. Before performing qPCR, extracellular DNA has to be removed to determine the amount of vital cells. Through the activity of DNase I, extracellular DNA is digested. When using this method, one has to directly focus their attention to reaction conditions, DNase I concentration, exposure time of DNase, and inactivation of DNase I, which can be properly inactivated by Proteinase K. Through DNase I/Proteinase K pretreatment, followed by qPCR, exclusively living cells were detected in the reference sample as well as in the natural drinking water biofilms (Villarreal et al. 2013). DNase I/Proteinase K treatment could be a promising alternative to PMA-qPCR technique.

#### RNA analysis

RNA (mRNA, pre-rRNA, and rRNA) can be used to quantify viable or recently active microbes (Cangelosi et al. 2010; Blazewicz et al. 2013). To quantify viable microbes via RNA analysis, RNA of high quality has to be extracted from the sample, which can be challenging (Rittmann and Holubar 2014). The short half-life of mRNA of minutes (Passow et al. 2018) in active cells can be seen as an advantage and a disadvantage at the same time. Specific metabolic responses of microbes can immediately be detected. On the other hand, extraction has to be performed fast or special sample preparation have to be considered, such as flash freezing in liquid nitrogen (Rittmann and Holubar 2014), or the application of stabilizing components like RNAlater (Passow et al. 2018). Compared to mRNA, rRNA has a half-life of hours (Karnahl and Wasternack 1992). Thus, when aiming for rRNA instead of mRNA to quantify active cells, extraction or stabilization of RNA can be performed slower compared to mRNA. Another advantage of using rRNA is that rRNAs are part of the ribosomes and thereby more protected as mRNAs. Besides rRNAs, ribosomes consist of ribosomal proteins, which among other tasks stabilize the protein synthesizing complex (Smith et al. 2008). As ribosomes (10^3^ to 10^5^ ribosomes per cell among different species) transcribe mRNAs and thereby synthesize new proteins, direct correlations with growth rate can be drawn (Kemp et al. 1993; Amann et al. 1995). However, rRNA is also present in dormant cell as well (Blazewicz et al. 2013). To circumvent this bias, precursor rRNA (pre-rRNA) can be targeted and quantified via qPCR (Cangelosi et al. 2010). RNA can be detected via microarrays, qPCR, 16S ribosomal RNA (rRNA) sequencing, and metatranscriptomics (Ozsolak and Milos 2011; DeAngelis et al. 2011; Geisen et al. 2015).

#### Genomics

If only the metagenomics data of a sample is available, iRep could be used to estimate genome replication rates from single-sample metagenomic data (Brown et al. 2016).

### Quantification of viable biomass by using physiochemical parameters

Physiochemical parameters can also be employed to estimate the viable amount of microbes in a population, such as:

1. Adenosine triphosphate (ATP)
2. Heat flows
3. Foam formation

#### ATP

Investigating biomass by measuring ATP is dependent on the fact that all viable cells contain ATP, whereas non-living particulate matter do not. The ratio of ATP to carbon in cells is fairly constant for living organisms even though it slightly varies from species to species. The energy-storing macromolecule ATP is only present in viable cells and disappears right after cell death (Helm-Hansen and Booth 1966). It was shown that the ATP content reflects the activity of anaerobic digestion (Chung and Neethling 1988). The ATP content of the biomass was determined through a luciferin-luciferase-mediated reaction. The generated luminescence intensity from the reaction was found to be proportional to ATP concentration in the assay solution and consistent results with 10% accuracy were achieved (Chang et al. 1981). ATP might be used also as a total activity indicator for anaerobic digesters. Adverse aspects are the limitation of distinguishing between the various population groups in a digester, but it could be used when working with pure cultures. Their results showed that the activity in a digester, measured as ATP concentration, responded quickly to changes in digester operation. Those changes have to be included when interpreting the results. Further, the ATP content of living cells is dependent on environmental conditions and reflects the activity of the cellular metabolism (Graça et al. 2005). The distinction between various species within a population cannot be performed via ATP measurements.

#### Heat flow

Another physiochemical marker for vital microbes is heat flow. Heat flow is an outstanding indicator for microbial activity, for the quantity of substrate consumption or metabolic product release. This can be measured by using isothermal calorimetry (IMC), which has already been proven to be an accurate method for monitoring microbial activity for in situ samples with very low detection limits. IMC provides a rapid real-time detection and monitoring of microbial growth and metabolism. Measurements of heat flow less than a microwatt, produced by 1 · 10^4^–1 · 10^5^ active bacterial cells, are possible to be detected with this non-destructive method (Braissant et al. 2010). The generated signal can be related to the number of present cells and their activity (Braissant et al. 2010). Investigations of lake and marine sediments have shown a linear relation between dehydrogenase activity assayed by using triphenyltetrazolium chloride (TTC) or iodonitrotetrazolium chloride (INT) and sediment heat production (Pamatmat and Bhagwat 1973; Pamatmat et al. 1981). Furthermore, a strong correlation between the ATP concentration and the heat production in the sediment was observed (Pamatmat et al. 1981). In 2003, a more recent study on lake sediments containing mixed communities of anaerobic, fermentative aerobic strains was performed (Haglund et al. 2003). They concluded that heat production followed the same trend as radiolabeled leucine and thymidine incorporation. Calorimetric chips are a promising area of IMC instrumentation (Van Herwaarden 2005). These chips have already been used to monitor bacterial growth correlated to heat (Higuera-Guisset et al. 2005; Maskow et al. 2006). Auspicious calorimetric techniques are enthalpy arrays (Torres et al. 2004), which detect molecular interactions including protein–ligand binding, enzymatic turnover, and mitochondrial respiration that reflect viable cells.

#### Foam formation

Foaming cultures indicate an augmented cell lysis, generated by an overload of lipids, proteins, and carbohydrates in the liquid phase (Kougias et al. 2014). Foam is a dispersion of gas bubbles in a liquid (Walstra 1989), where the biggest volume consists of gas surrounded by a thin liquid film (Mollet and Grubenmann 1999). In bioprocesses, foaming can be caused by surface-active compounds, VFLs, lipids, and proteins. Two groups of surface-active substances are closely related to foam formation: surfactants and biosurfactants (Ganidi et al. 2009). VFAs, oil, grease, detergents, and proteins are examples of surfactants (Moeller et al. 2012). Biosurfactants are naturally produced substances through microbial activity in the bioreactor (Ganidi et al. 2009), such as hydroxylated and cross-linked fatty acids, glycolipids, lipopolysaccharides, lipoproteins–lipopeptides, phospholipids, and the complete cell surface (Saharan et al. 2012). Volatile fatty acids like formic acid, acetic acid, propionic acid, butyric acid, isobutyric acid, valeric acid, and 3-methylbutanoic acid are potential intermediates of biogas production (Moeller et al. 2012). Due to their hydrophobic character, lipids have the tendency to diffuse to the surface (Berg et al. 2013), and as a result, lipids incline foam formation. Not only lipids can enhance foam formation but all cell lysis and cell metabolism-related compounds can contribute to foam formation. Further, gases can promote foam formation (Subramanian and Pagilla 2015). Foaming caused by CO_2_ seems to be spontaneous (Devereux and Lee 2011), whereas bubble nucleation in presence of CH_4_ requires an initiator (mixing) for foam (Blatteau et al. 2006; Subramanian and Pagilla 2015). Changes in microbial population of anaerobic digestors, fed with agro-industrial wastes, before and after foam formation were studied. Interestingly, no archaea was found to be associated to the foaming event, but some archaeal species increased their abundance corresponding to foam formation (Kougias et al. 2014). Foaming is an indicator for cell lysis during fermentation, but due to complexity of foam formation, it is not possible yet to correlate foaming intensity to the amount of dead cells. However, foaming could in some cases be indirectly measured via quantification of the specific lysing rate via the quantification of specific process parameters such as specific amino acid ratios (Bernacchi and Herwig 2017). Correlating foaming intensity to the amount of dead cells would be a useful tool in biotechnology.

## Discussion—quantification of live and dead biomass

The assessment of microbial viability during cultivation is essential to be able to monitor or improve the targeted parameters, such as productivity. Microbial viability can be monitored via staining methods followed by microscopic visualization or FACS, molecular-based methods, or physiochemical parameters. Staining methods like LIVE/DEAD BacLight^®^ bacterial viability kit, BONCAT, or MAR have the advantage that organisms can be visualized. Via the viability kit, dead cells in a population are assessed through disrupted cell membranes. However, the BONCAT technique enables a specific detection of transcriptional active cells (Hatzenpichler et al. 2014) since newly synthesized and labeled proteins are detected. MAR is limited to single-cell activity measurements, although it is based on a short-term incubation of radioactive-labeled substrate (Nielsen et al. 2003). If cells cannot be visualized, molecular-based methods like PMA/qPCR, DNase I/Proteinase K, RNA analysis, and metagenomics data could be used to determine the viability. Other methods that give insights toward viability status of a population are physiochemically based methods like ATP and heat flow measurements. The detection of the macromolecule ATP via performing an assay could be used to determine active cells (Helm-Hansen and Booth 1966; Chang et al. 1981). A more precise technique to determine viability is the measurement of the heat flow produced by microbes. It has been already proven that heat flow especially isothermal calorimetry (IMC) (Braissant et al. 2010) and enthalpy arrays (Torres et al. 2004) are an accurate method to monitor microbial activity for in situ samples with a very low detection limits. Compared to heat flow measurements techniques, the correlation between foaming events and viability status of the culture is mostly unclear.

## Quantification of liquid-phase substrates and products

In nature, microbial cells are exposed to a wide spectrum of potential substrates, many of which they could utilize simultaneously, serially, or the organisms re-assimilate metabolic end products (Martinez-Porqueras et al. 2013). Especially in biotechnology, tracing how fast substrates are utilized and converted into products is critical when assessing the efficiency of the metabolism of a microorganism.

## Analytics for liquid-phase substrate and product quantification

There are several applied methods to monitor substrate uptake and track product formation. When investigating liquid-phase substrates and products, the medium characteristics in which the targeted compound is dissolved have to be taken in account. The opacity, viscosity, and corrosiveness of the working medium can interfere with quantification techniques. Those features have to be elucidated before starting the quantification and adapted to specific medium features. Liquid substrates or products can be quantified or even identified with the following techniques:

1. Chromatography
2. Mass spectrometry
3. Spectroscopy
4. Assay kits
5. Stable isotope probing

### Chromatography

Chromatography is a chemical technique that is primary used for the separation of components of a mixture. The principal of separation is based on the interaction between the analyte and the mobile and the stationary phase. The separation method and the downstream detector depend on the investigated component. Liquid chromatography (LC) can be divided into thin-layer chromatography and column liquid chromatography (high-performance liquid chromatography (HPLC) and ultra-performance liquid chromatography (UPLC)). HPLC allows a faster separation of the investigated analysts as LC (Gey 2015a). Compared to HPLC, the separation process in case of UPLC is performed with approximately 1000 bar. This leads to an improved resolution and sensitivity, as the peaks in the chromatogram became thinner. Further, the operation speed is increased (Nováková et al. 2017). Commonly used techniques to quantify metabolized or produced components of anaerobic microbes are LC and HPLC. HPLC allows the separation of amino acids, peptides, proteins, lipids, vitamins, organic acids, or bases, e.g. within the sample. The combination of HPLC and MS admits an accurate determination of the analyte (Nollet and Toldrá 2012). Using HPLC equipped with an autosampler, a quaternary pump, a UV detector, and an Aminex HPX-87H (300 × 7, 8 mm) column, short-chain fatty acids (formic, acetic, butyric, propionic acid) could be measured during anaerobic digestion processes and their effect toward CH_4_ production (Wagner et al. 2011). Further, the concentrations of dissolved free taurine and dissolved free amino acids could be determined via HPLC fitted with an autosampler, a quaternary pump, a column oven and a fluorescence detector (Clifford et al. 2017). After performing a supercritical fluid extraction (SFE), bacterial respiratory quinone (RQ), bacterial phospholipid fatty acid (PLFA), and archaeal phospholipid ether lipid (PLEL) from anaerobically digested sludge can be analyzed. Bacterial RQ were determined using UPLC (Hanif et al. 2012). To identify and quantify liquid components in microbial cultures, mass spectrometry (MS) could be coupled to LC or HPLC.

### Mass spectrometry

MS enables the measurement of atoms or molecules within a sample. A mass spectrometer consists of a sample inlet, ion source, mass analyzer, detector, control unit, and evaluation module. MS is mainly used to elucidate structures of organic molecules. The sample gets converted into a positively or negatively charged gaseous ion by using an ion source followed by ion separation and detection in the mass analyzer unit based on their mass-to-charge ratio (*m*/*z*). To avoid collusions of ionized particles, high-pressure vacuum (10^−4^ mbar) is applied in the device (Gey 2015b). Depending on the sample different ion sources, mass analyzer and detectors can be combined. In principle, ionization can be divided into gaseous (electrospray ionization (ESI), chemical ionization (CI), field ionization (FI)) and desorption (field desorption (FD), fast atom bombardment (FAB), matrix-assisted laser desorption/ionization (MALDI)) techniques and soft and hard ionization. Hard ionization methods (electron ionization (EI)) cause several ion fragmentations, whereas soft ionization methods (CI, FI, ESI, FD, FAB, atmospheric pressure chemical ionization (APCI), atmospheric pressure photoionization (APPI), and MALDI) induce no or hardly any fragmentation of molecules. The most commonly used mass analyzers are magnetic sector mass spectrometers (MS/MS), quadrupole mass spectrometers (QMS), time-of-flight mass analyzers (TOF), trapped-ion mass analyzers (IT), and quadrupole ion traps (QIT). MS/MS provides high reproducibility, resolution, and sensitivity. Organic MS analysis, accurate mass measurements, and isotope measurements can be performed with this set-up. Although this mass analyzer is commonly used, it is more expensive than other mass analyzers; also, it is not well suited for MALDI. QMS has a good reproducibility and is relatively small and low cost, although the resolution is limited and the combination with pulsed ionization (MALDI) is not recommended. This analyzer is compatible with MS/MS, GC/MS, and LC/MS. TOF is known to be a fast MS analyzer and well suited for MALDI, pulsed ionization methods in general, and fast GC/MS systems. IT has the highest recorded mass resolution. However, this device requires strict low-pressure conditions. Compatible ionization techniques are MALDI and ESI with high mass analytes. QIT has a high sensitivity but poor quantitation. Applications are ion chemistry and target compound screening. Compatibility is ensured with GC/MS, LC/MS, and MS/MS. Toward separation, ion detection is executed. Established detectors are photomultiplier tube (PMT), electron multiplier tube (EMT), and Faraday cup (FC) (Brunnée 1987). Faraday cup detectors are mostly used in IRMS devices (Evershed et al. 2006; Chartrand et al. 2007; Schulze-Makuch et al. 2011). Electrospray ionization (ESI) and matrix-assisted laser desorption/ionization (MALDI) coupled to time-of-flight (TOF) analyzer are the most appropriate ionization methods for biomolecules like peptides, proteins, nucleic acids, oligosaccharides, and lipids (De Hoffmann and Stroobant 2007). For the ionization of steroids, amino acids, vitamin D, fatty acids, and fullerenes, ESI can be used (Wilson and Wu 1993). MALDI is used for the ionization of following biomolecules (Duncan et al. 2016), lipids (Wang et al. 2015), carbohydrates (Harvey 2003), drugs including drug metabolites (Buck and Walch 2014), hormones (Gao et al. 2015; Yi et al. 2015), and nucleotides and nucleosides (Gao et al. 2012). Further, MS (ESI, MALDI) could function as a tool to study enzymatic reactions (Liesener and Karst 2005). The combination of chromatography and mass spectrometry enables a threshold of investigated compounds within nanogram and femtogram range (Gey 2015b). Some application areas are listed below.

#### LC/ESI-QMS

Mass spectrometric analysis of large biomolecules is preferentially investigated by using ESI-MS, which is predominantly coupled with LC. Since a QMS detector was used, the method is named LC/ESI-QMS. ESI-MS has a broad applicability such as analyte quantification, structure determination of biomolecules and protein–ligand interaction studies. Also, the competitive consumption of two substrates was investigated of an archaeal glycogen synthase by using ESI-MS (Zea et al. 2003).

#### LC/MALDI-TOF-MS

LC/MALDI-TOF-MS is commonly used in detection and verification of carbapenemase production in anaerobic bacterium *Bacteroides fragilis*, which belong to the beta-lactamase protein family and inhibits most beta-lactam-based antibiotics (Johansson et al. 2014).

#### HPLC/APCI-MS

HPLC combined with MS with positive ion atmospheric pressure chemical ionization mass spectrometry (APCI-MS) could be used to investigate of intact glycerol dialkyl glycerol tetraethers (GDGTs) in archaeal cell. Molecules could function as biomarkers to detect archaeal cells (Hopmans et al. 2000).

#### UPLC-UV-ESI-MS/MS

The relative abundance of 2-amino-1-methyl-6-phenylimidazo(4,5-b)pyridine (PhIP) and PhIP-M1 in cultures of the strict anaerobic gut microbe *Eubacterium hallii* were analyzed with UPLC-UV-ESI-MS/MS (Fekry et al. 2015). The separation was performed with UPLC, the ionization with ESI, and the mass analysis with MS/MS.

### Spectroscopy

Spectroscopy, particularly infrared and Raman spectroscopy, can be applied to monitor various metabolites during cultivation (Kornmann et al. 2003). Infrared sensors are commonly used in biotechnology. When monitoring the consumption of a substrate or production of a product in the liquid phase, NIR, MIR, and FIR spectroscopy methods could be applied. Near-infrared spectroscopy (NIRS) was used for simultaneous prediction of exopolysaccharide (EPS; 0–3 g/L) and lactic acid productions (0–59 g/L), and lactose (0–68 g/L) concentration in supernatant samples from pH-controlled batch cultures of *Lactobacillus rhamnosus* RW-9595 M (Acedo et al. 2002). Linoleic acid, oleic acid, and ammonia were measured in fermentation broth via an inline NIR of following microbes: *E. coli*, *Pichia pastoris*, *Streptomyces toxitricini*, and *Aspergillus niger* (Tiwari et al. 2013). Methanol concentrations were tracked by applying an online MIR sensor while performing a fermentation with *P. pastoris* (Schenk et al. 2007). Online Raman application could be used to determine starch, dextrins, maltotriose, maltose (Gray et al. 2013), glucose, and ethanol (Shaw et al. 1999) concentrations in the fermentation broth of *S. cerevisiae*. During fermentations of *E. coli*, online Raman was used to determine glucose, lactate, formate, acetate, and phenylalanine (Lee et al. 2004). Online spectroscopy to track substrate uptake is a useful tool to monitor various metabolites during fermentation*.*

### Assay kits

Assay kits could be used to determine the uptake of the employed substrate. For instance, uptake of starch, mono-, di-polysaccharides, alcohols, and organic acids could be tracked by using designated assay kits (Megazyme Inc., USA; www.megazyme.com). For quantifying the amount of residual substrate or produced product, ELISA could be examined (Neuhaus et al. 2015).

### Stable isotope probing

SIP techniques could be used to illustrate and track substrate uptake and metabolic processes through labeling of specific biomarkers (Musat et al. 2012). SIP approaches mainly use stable isotopes, such as ^13^C, ^15^N, or ^18^O. ^13^C-tracers are widely used to asess the quantity of carbon flux. SIP techniques are predicated upon the incorporation of labeled substrates into DNA (DNA-SIP; Radajewski et al. 2000), RNA (RNA-SIP; Manefield et al. 2002), proteins, or phospholipid fatty acids (PLFA-SIP; Middelburg et al. 2000).

#### DNA-SIP and RNA-SIP

The incorporation of labeled substrate with DNA-SIP and RNA-SIP approach could be visualized via isotope ratio mass spectroscopy (IRMS) or at single cell level by *FISH-MAR*, *FISH-SIMS* (Biddle et al. 2006), *FISH-Raman* (Haider et al. 2010), and *NanoSIMS* (Lechene et al. 2006). Also, unstable isotopes (^14^C, ^3^H, ^35^S, ^33^P, ^32^P) are commonly used in research to study the metabolism of microbes. *FISH-MAR* can be used for the specific detection of the microorganism (FISH) and monitor the incorporation of labeled substrate, such as^14^C, ^3^H, ^32/33^P (Lee et al. 1999), and ^35^S (Vila et al. 2004) into intracellular storage compartments. This technique is limited by the availability and affordability of radioactive-labeled substrates (Nielsen and Nielsen 2010). Further, microbes that assimilate radioactive-labeled substance cannot be discriminated from active ones via the application of MAR (Musat et al. 2012). *FISH-SIMS* was applied to identify the metabolism of two uncharacterized archaea, which naturally present in the subsurface of marine sediments by studying their isotopic carbon (Biddle et al. 2006; Musat et al. 2012). *FISH-Raman* is applicable to investigate the metabolic function of microbial cells (Haider et al. 2010). *NanoSIMS* could be used as a sole approach or in combination with others, like FISH, SIMSISH, EL-FISH/HISH-SIMS. The N_2_-fixation of a bacterial symbiont of a shipworm was intensively studied with NanoSIMS (Lechene et al. 2006). Microbial cells could be identified by using FISH or halogens (bromine, fluorine, or iodine) bonded directly to oligonucleotide probes that bind specifically to rRNA genes of the targeted organism (Musat et al. 2012). The usage of SIMSISH (iodine-labeled oligonucleotide probe) is favored, when the permeabilization of cell wall is barely realizable (Amann and Fuchs 2008). EL-FISH (Behrens et al. 2008)/HISH-SIMS (Musat et al. 2012) was based on bromine- and fluorine-labeled tyramines in oligonucleotide probes. This technique was used to study and identify rare microbes involved in N_2_ fixation in anoxic layers of lake sediments (Halm et al. 2009).

## Discussion—quantification of liquid-phase substrates and products

The quantification of liquid-phase substrate conversion to product is essential when studying physiology a microorganism. Liquid substrate consumption and product formation during an anaerobic cultivation can be investigated via chromatography (LC, HPLC, and UPLC). Chromatography followed by mass spectrometry (MS) analysis that enables the identification and measurement of a broader range of liquid compounds down to nanograms and femtograms. Most analyses performed with biomolecules are performed with soft ionization methods like ESI, MALDI, or APCI. The advantage of using soft ionization methods is due to that only a little amount of molecule fragmentation is induced, which allows the conservation of molecular structures (Gey 2015b). The most commonly used mass analyzer (detector) is MS/MS due to its high reproducibility, resolution, and sensitivity. The broad operational area of MS/MS mass analyzer is reflected in the acquisition costs since MS/MS is more expensive than others. Although MS/MS is commonly used, it is not compatible with MALDI, whereas TOF is compatible with MALDI. Also, IT could be executed with MALDI and ESI investigating high mass analytes (Brunnée 1987). Depending on the hypothesis and the experimental framework, different MS set-ups including ionization method, mass analyzer, and detector can be combined. If liquid substrate uptake and metabolite production of a culture during cultivation should be investigated, infrared and Raman spectroscopy could be applied (Kornmann et al. 2003). Compared to spectroscopy, assay kits or ELISA are a much more cost-efficient technique to measure substrate uptake (Neuhaus et al. 2015), although the measurement has to be performed offline.

SIP techniques employ the possibility to illustrate and monitor substrate uptake during metabolic processes. Through stable isotope labeling (^13^C, ^15^N, or ^18^O) of specific biomarkers, substrate uptake and conversion performed by microbes can be illustrated and tracked (Musat et al. 2012). Also, unstable isotopes (^14^C, ^3^H, ^35^S, ^33^P, ^32^P) are commonly used in research to study the metabolism of microbes for instance. Compared to the half-life time of ^14^C (5.73 × 10^3^ years), radioactive decay occurs much faster for ^3^H (12.3 years), ^35^S (87.4 days), ^33^P (25.4 days), and ^32^P (14.3 days) (IAEA 2011). When working with radioactive isotopes, not only half-time is of great importance but also the rate of radiation that is emitted, given in Bequerel (Bq radioactive decay [s^−1^]), the emitting distance, and the estimated damage to the body from absorbed radiation, measured in Sievert (Sv [J kg^−1^]). The annual worldwide exposure to natural radiation sources is being expected to be in the range of 1–10 mSv, while the present estimated central value is expected to be 2.4 mSv (United Nations 2000). Including also the civilizing radiation exposure of approximately 2 mSv/year, people living in industrialized countries have to cope with approximately 4.4 mSv/year. Radiation doses below 1 Sv show no symptoms or occasionally mild nausea. Values from 8 Sv onwards have a lethal effect for human beings within 30 days. There are also correlations between radioactive contamination, increased tendency to get cancer, and germline mutations (Lottspeich and Engels 2012). When working with unstable isotopes, people have to be aware that already little amounts of incorporated radioactive material could drastically increase the personal Sv value. After working with radioactive material, it has to be disposed of properly. In Austria, radioactively contaminated waste has to be collected and stored at Nuclear Engineering Seibersdorf GmbH. The costs for the disposal are divided into the transport to the permanent repository (2.2 € km^−1^), wage (124 € h^−1^), the radioactive waste (biological origin 152 € kg^−1^), and the sales tax (Nuclear Engineering Seibersdorf 2018). Working with radioactive substances should only be performed under highest safety precautions to not contaminate living organisms or the environment.

## Quantification of gaseous substrates and products

When discussing substrates and their utilization by microorganisms, also the substrate availability for the organism has to be considered. Substrate availability is crucial, especially in case of gaseous substrates as some of them could exhibit a low solubility in liquid media, like H_2_ and CO. Gaseous substrates might not become biologically available because of by-product reactions such as complexation of trace elements (Morse and Luther 1999). The solubility of gaseous substrates can be increased by applying pressure and performing the experiment at low temperatures (Follonier et al. 2012).

## Gas analytics for substrate and product quantification

Most anaerobes are able to generate biotechnologically important gaseous end products during their metabolism. Biological gas production of gases can be monitored and quantified through:

1. Gravimetric determination
2. Volumetric based determination
3. Pressure change
4. Gas chromatography
5. Infrared sensors

### Gravimetric determination

If the production of gaseous end products is associated with the production of water, the quantification of gas in a closed batch cultivation device can be determined through a weight increase. In this case, gas consumption has to be compensated via isobaric determination or during continuous gas conversion. The production of CH_4_ in closed batch cultivation setting can be performed by autotrophic, hydrogenotrophic methanogens in sequential rounds of gassing, gas consumption, and gravimetric determination of mass increase at isobaric conditions (Taubner and Rittmann 2016). The principle of this method is based on the stoichiometry of autotrophic, hydrogenotrophic methanogenesis shown in Eq. 3.

3 $$ {4\mathrm{H}}_2+{\mathrm{CO}}_2\rightarrow {\mathrm{CH}}_4+{2\mathrm{H}}_2\mathrm{O} $$

The production of 1 mol of CH_4_ through the conversion of 5 mol of gaseous substrates (H_2_ and CO_2_) is accompanied by the production of 2 mol of water (H_2_O). CH_4_ production is therefore related to the production of H_2_O, which corresponds to an increase in weight. The reaction stoichiometry can be used to quantify the volumetric CH_4_ evolution rate (MER) by measuring the volumetric H_2_O evolution rate (WER) (Taubner and Rittmann 2016). Similar gravimetric determination of gas to liquid conversion can be used in continuous culture systems that use autotrophic, hydrogenotrophic methanogens. In an online-controlled continuous culture system, *M. marburgensis* produced CH_4_ and H_2_O according to Eq. 3. It was found that WER could serve as an online variable to quantify MER at high gas flow rates (Rittmann et al. 2012).

### Volumetric-based determination

If gaseous substrates are supplied during a cultivation of anaerobes in a bioreactor set-up, either fed-batch or continuous mode, off-gas measurements could give insights in gas consumptions or gaseous product formation. This technique can be used if the gas outflow does not equate the gas inflow. A flow meter is a precision instrument that measures the rate of gas flow in a pipe. The gas flow can be investigated via measurements of mass flow, velocity flow, differential pressure, and positive displacement. *Mass flow meters* measure the rate of mass flow through a conduit. It is important to note that the exact gas compositions has to be known to be able to determine the gas flow. The application of mass flow meters is recommended to quantify pure gases but difficult to apply for gas mixtures. If the gas flow of a mixture is investigated via a mass flow meter, the exact gas composition has to be known. Examples include Coriolis flow meters and thermal mass flow meters. *Coriolis flow meters* are based on the deflection force caused by fluid moving through a vibrating tube. Coriolis mass flow meters are currently being used in many industrial areas, e.g. chemical, petroleum, pharmaceutical, food, beverage, and paper industry (Anklin et al. 2006) or during thermophilic anaerobic digestion to determine the gas flow of a 40 m^3^ semi-continuous operating bioreactor (Espinosa-Solares et al. 2006). Since the Coriolis flow meter measures mass flow and not volume flow, the flow meter is often used near the lower detection limit. Another positive property of this technique is the independency of fluid properties. The higher the mass flow of a gas is, the better is the detection, although there is an upper limit for flow speed that is encountered to be approximately half the mach number of the gas. Further, it is recommended to install a Coriolis flow meter at a high pressure side, unlike many other flow measurement technologies (Anklin et al. 2006). *Capillary thermal mass flow meters* (CTMF) measure the mass flow based on heat transfer from a heated element. The gas flows through a very narrow tube, on which heating and temperature-sensing windings are attached. The gas flow is heated via the heat winding. The specific heat capacity of the gas and the temperature difference between the downstream, the upstream temperature sensor, and the specific heat capacity of the gas will then be used to deduce the mass flow. Flow ranges are from 0 to 100 L min^−1^, 0 to 3 mL min^−1^, or for special bypass designs (1000 m^3^ h^−1^). These meters can be operated up to 200 bar or even, in some cases, 300 bar pressure. Operating temperature ranges from 0 to 65 °C. Most CTMFs are commonly applied to low flows of clean dry gases above their dew points (Baker 2000; Viswanathan et al. 2002). Thermal and Coriolis mass flow meters can be operated maximum at 300 and 400 bar, and maximum applied in a temperature range between 0 to 300 °C and − 240 to 426 °C. The accuracy of thermal flow meters has a deviation of 1% to full scale and Coriolis flow meters achieve a precision of 0.1–0.3% in terms of rate. In case of thermal flow meters, the pipe run has to be short, whereas the applications of Coriolis flow meters do not have any restrictions. The relative pressure loss is low in case of thermal mass flow meters and low to middle in case of Coriolis mass flow meters (Green and Perry 2008).

*Velocity flow meters* measure fluid velocity. A vane anemometer, for instance, could be used for gas-velocity measurements in the range of 0.3 to 45 m s^−1^. Flow meters which are based on velocity are electromagnetic, propeller, turbine, ultrasonic Doppler, ultrasonic transit time, and vortex flow meters (Green and Perry 2008). *Ultrasonic flow meter* measurements are based on the slight difference in time taken for an ultrasound wave to travel upstream rather than downstream. Thus, waves are detected each way, their time of transit is measured, and this difference can then be correlated to the speed of the flow. This style of meter is immensely accurate but extremely expensive. Pressure and temperature measurement are required as well. Ultrasonic flow meters are mostly used for industrial purposes such as gas industry (Baker 2000). *Vortex flow meter* has a bluff object that is placed in the flow path, generating vortices. The relation between the mean flow velocity and the vortex frequency generated by bluff bodies is measured. A minimum flow rate, temperature, and pressure compensation is needed to produce vortices. Commonly, the vortices are measured via pressure sensor in the pipe wall or inside the bluff body. Due to the low sensitivity of pressure sensors, strong vortices have to be generated by large bluff bodies. An alternative detector would be an ultrasonic barrier behind the bluff object vertical to the pipe axis and the bluff body (Hans and Windorfer 2003). A vortex flow meter, Endress-Hauser Prowirl 72 (Baker 2000), was used to determine biogas in an AnaEG anaerobic bioreactor, which produces an average of 30 m^3^ biogas/m^3^ of raw palm oil mill effluent (Tabassum et al. 2015). Ultrasonic and vortex flow meters can be used maximum at 400 and 100 bar, and maximum applied in a temperature range between − 40 to 340 °C and − 200 to 426 °C. The accuracy of vortex flow meters is increased (0.5–2%) respectively to rate compared to ultrasonic flow meters (0.5–5%). Ultrasonic flow meters need a long pipe run, whereas vortex flow meters are restricted to a short pipe run. The relative pressure loss is low in case of ultrasonic flow meters and middle in case of vortex flow meters (Green and Perry 2008).

*Differential pressure flow meters* measure the pressure change (pressure drop) over a special flow element, an obstruction inserted in the flow path. Gas density is affected by temperature and pressure. Therefore, differential pressure flow meters are often additionally equipped with integral temperature and absolute pressure measurement devices (Green and Perry 2008). Common types of flow elements are orifice plates, flow nozzles, venturi tubes, and averaging pitot tube. The *orifice plate flow meter* is the most common differential pressure flow meter and is frequently used for natural gas measurement (Baker 2000) including land field gases, such as CH_4_ and CO_2_ (Tolaymat et al. 2010). It is made of a metal plate with an orifice that is inserted between flanges with pressure tappings formed in the wall of the pipe. Almost any single-phase Newtonian flow can be measured with an orifice plate flow meter (Baker 2000). A *flow nozzle* consists of a short cylinder followed by a widened section (funnel-like structure). Two pressure sensors detect the alteration of the gas flow, the upstream pressure tap (higher pressure) is located approximately one pipe diameter before the nozzle inlet face and the downstream pressure tap (lower pressure) about 1/2 pipe diameter from the inlet face. The standard *Herschel-type venturi meter* consists of three sections, a short straight tubing (throat section), which is connected at both ends to conical pipe lines. Pressure taps are positioned at the inlet section (conical) and at the throat section. *Averaging pitot tubes* produce a pressure differential that is based on multiple measuring points through the flow path. Pitot tubes are useful when a system has no permanent gas flow sensors. Orifice plate, flow nozzles, venturi, and averaging pitot tube flow meters can be used maximum at 600 (orifice and multivariable flow transmitter 275), >70, 600, and 600 bar, and maximum applied in a temperature range from −20 to 1260 °C (orifice and multivariable flow transmitter 540 °C), maximum 540 °C, − 20 to 1260 °C, and − 29 to 1300 °C. The accuracy of orifice plate flow meters lies between 0.5 and 2% respectively to rate, which also counts for flow nozzles. Under optimal conditions, venturi flow meters have an accuracy between 0.5 and 1.5% (rate); averaging pitot tube flow meters imply a precision of 1% respectively to rate. Orifice flow meters need a long pipe run; for the other flow meter types, this was not investigated yet. Venturi and averaging pitot tube flow meters show a low relative pressure loss, whereas orifice plates and flow nozzles have a middle relative pressure loss (Green and Perry 2008). *Positive displacement meters* require fluid (liquid or gas) to mechanically displace or move components that hinder the fluid flow. Thereby the volumetric flow is measured at the operating temperature and pressure. The high accuracy of this method leads to a broad application field including oil and gas industry. The advantage is that the flow meter can be used independent of the gas composition. Positive displacement meters being are gradually being replaced by other modern approaches such as turbine, ultrasonic, and Coriolis flow meters (Crabtree 2009). An advantage of this method is the independency of the gas composition, therefore it is well suited to determine the gas flow of gas mixtures. The operation of a drum-type gas meter is based on the displacement principle to elucidate the gas flow. During fed-batch cultivations of *Methanobacterium thermaggregans*, the gas flow was experimentally determined via a TG3 plastic drum-type gas meter (Mauerhofer et al. 2018). This device can be operated in offline and online mode. The pressure inside the bioreactor and the off-gas temperature has to be monitored and integrated into the determination of the off-gas flow. Drum-type gas meters show an accuracy of ± 0.5% across the full flow rate range and can be operated between 50 mbar (plastic casings) and 500 mbar (stainless steel casings) within a temperature range of − 10 to 80 °C. Depending on the size, 1 to 18,000 L h^−1^ can be investigated.

### Pressure alteration

Another method to determine biologically consumed or produced gases is the detection of pressure changes in the cultivation device. Pressure in the cultivation vessels or the bioreactor can increase or decrease, depending on the cultivated organism, applied feed and undergoing conversions. When cultivating H_2_ producers, pressure levels in the cultivation vessel increases due to the production of H_2_ out of liquid substrates, e.g. glucose (Fig. 4a). Numerous fermentation experiments with microbes have shown that, under optimal conditions, the oxidation of glucose will at best result in the formation of four molecules of H_2_ per molecule of hexose, in addition to acetate and CO_2_ production (Verhaart et al. 2010; Rittmann and Herwig 2012). Biological H_2_ production is associated with a pressure increases in the closed batch cultivation vessel during gas production from liquid compounds or during CO oxidation by carboxydotrophic H_2_-producing microorganisms (Rittmann et al. 2015a). However, a pressure drop occurs in the cultivation vessel when, e.g. autotrophic, hydrogenotrophic methanogens are cultivated (Fig. 4b) (Taubner and Rittmann 2016). The pressure difference measured over time is then correlated back to produced CH_4_ and consumed gas by using the assumption set the authors have formulated. Indirectly, microbial growth or metabolic end product formation is indicated through a pressure change in a closed batch system (Miller et al. 1988; Follonier et al. 2012; Keymer et al. 2013; Taubner and Rittmann 2016). However, gas leakage or reaction with other medium compounds must be avoided.

### Gas chromatography and infrared sensors

Gas chromatography is a biophysical technique that is used for the detection of gaseous components of a mixture. The principle of separation and the downstream detector is dependent on the investigated component. Metabolized or produced gaseous components of anaerobic microbes are commonly measured with gas chromatography (GC) (Gey 2015b). Gas chromatography is used to analyze thermally stable and volatile compounds, or compounds that can be made volatile.

#### Operation principle

The basic operation principle of a GC includes the evaporation of the sample in a heated inlet port (injector), separation of the component in a column, and the detection of each component by a detector. First, a certain volume of the sample with proper pressure and temperature has to be transferred to the GC. Sampling for offline determination of head space gas compositions can be simply performed manually with gas-tight syringes (Keymer et al. 2013) or automatically with a gas injection control unit (Joint Analytical Systems GmbH, Germany). After transferring the sample to the GC, it is injected into a steam of the carrier gas, which should be an inert gas or not react with the investigated components in the samples.

#### Online GC systems and μ-GC (portable)

Online GC systems need further equipment and appliances to ensure a safe and efficient coupling between the bioreactor system and the gas chromatograph. In case of an online GC, inlets have to be connected to the off-gas stream of the bioreactor (Ward et al. 2011). The sampling port has to consist of a sample loop and the pressure has to be set by a back pressure regulator (Miller et al. 1988; Nelson et al. 1991). At incubation pressures higher than 10 bar, an additional reservoir can be installed between the GC and the reactor to decompress the sample in advance to injection (Nelson et al. 1991). A micro-GC (μ-GC) is a small, portable GC system that can be operated in remote environments, as it is equipped with a battery. In contrast to regular GC, the measuring time for each sample is significantly reduced. μ-GCs are available with up to two different and parallel columns and a thermal conductivity detector (Ward et al. 2011; Krümpel et al. 2016).

#### Carrier gases

Commonly used carrier gases are helium (He), N_2_, argon (Ar), and H_2_. Carrier gases have to be chosen respectively to the requirements of used column and detector and the target gas. In general, carrier gases differ in their separation efficiency and speed. The shortest time of analysis can be accomplished by applying H_2_ as carrier gas due to its low viscosity. Although He provides the best peak resolutions for many applications, it is commonly used as a carrier gas. Due to a possible destruction of columns, only carrier gas with high purity should be used (Gey 2015b).

#### Columns

The flow of this carrier gas transports the sample to the column (packed or capillary column), which is installed in a thermostatically controlled oven. Packed columns consist out of particles (150–250 μm), which are covered with a liquid mobile phase. At room temperature, the mobile phase has the consistence of a viscose oil or wax. As packed columns have low separation efficiency (Gey 2015b), nowadays mainly capillary columns are used. The capillary column consists out of a fused-silica material (quartz glass) that is coated with polyimide (thin polymer). Generally, the inner area of the column is chemically modified with a liquid stationary phase. Different types of capillary columns are available as they are wall coated open tubular column (WCOT), porous layer open tubular column (PLOT), and support coated open tubular column (SCOTT) (Gey 2015b). As components differ in their degree of interaction with the stationary phase in the column, they move with distinct velocities, which lead to the separation of components. The eluted components are then transported via the carrier gas from the column to a suitable detector. Depending on the components to be measured, different detectors can be applied.

#### Detectors

The most commonly used detectors are flame ionization detectors (*FIDs*), thermal conductivity detectors (*TCDs*), electron capture detectors (*ECDs*), and alkali flame ionization detectors—also called nitrogen/phosphorous detectors (NPDs), flame photometric detectors (FPDs), and photo ionization detectors (PIDs). Also, detectors need auxiliary gases for their operation. FID, NPD, and FPD require a mixture of synthetic air and H_2_ to create a flame. The ECD runs on N_2_ and/or on a CH_4_ mixture in Ar. Upon the usage of a TCD, the same gases are applied for make-up gas (flushing gas of the detector to prevent contaminations), detector gas, and carrier gas. As FID, TCD, and ECD are the most commonly used detectors for GC, only those three will be discussed in detail.

*FID* can detect most carbonic compounds, except formic acid and formaldehyde. Substances like He, NH_3_, CO, CO_2,_ H_2_S, H_2_O, O_2_, N_2,_ N_2_O, NO, and NO_2_ give little or no response in the FID (McNair and Miller 2011). In this case, H_2_ and synthetic air are required for the detection procedure. Both gases should not have any contaminations of carbon. The burning of H_2_ alone results in radical formation. However, the combustions of analyst that contain C–C or C–H compound lead to radical and ion generation. The eluted substance from the column is being burned by H_2_/air mixture (Gey 2015b). Within the H_2_-rich area of the flame, all carbonic compounds get reduced to CH_4_, whereas radical formation occurs in the O_2_-rich area of the flame due to oxidizing conditions. Radicals can react with induced O_2_ compounds to CHO^+^. Those ions get drawn by the collector (negatively charged) and generate the detector signal. GC/FID has been described as a useful and rapid analytical method for monitoring acetone, some alcohols, and VFAs in samples from anaerobic processes and from the environment. Headspace analysis of acetone, methanol, ethanol, isobutanol, *n*-butanol, acetic acid, propionic acid, isobutyric acid, butyric acid, isovaleric acid, valeric acid, crotonic acid, and caproic acid indicated good linearity, precision, and low detection and quantification limits. Thus, GC/FID can be applied to monitor the status of wastewater anaerobic treatment systems (Adorno et al. 2014). However, investigations have been shown that thermal desorption-GC (TD-GC) technique is better suited to determine VFAs (Ullah et al. 2014).

A GC equipped with a *TCD* allows the analysis of permanent gases, such as H_2_, N_2_, O_2_, CO, CH_4_, and CO_2_. The TCD measures the changes in conductivity of the sample which is eluted from the column. Gases that can be used for TCD are H_2_, He, Ar, and N_2_. The TCD compares the thermal conductivities of two gas flows—carrier gas and carrier gas including the sample components (column effluent). This detector contains an electrically heated filament out of tungsten or platinum that has a temperature-dependent resistance. The filament temperature is kept constant while alternate streams of carrier gas and column effluent pass over the filament. The changes in conductivity due to the presence of an analyte lead to a heat accumulation and the electric resistances increases. The power that is required to keep the filament temperature constant is being measured. This power difference is recorded. If He or H_2_ is used as carrier gas, the sample leads to a reduction of the thermal conductivity, respectively to their high conductivity approximately 1500 J cm^−1^ s^−1^ K^−1^ for He and 1800 J cm^−1^ s^−1^ K^−1^ for H_2_, whereas the application of N_2_ and Ar as a carrier gas would lead to an increase of thermal conductivity due to its low conductivity of approximately 200 J cm^−1^ s^−1^ K^−1^ for argon and 250 J cm^−1^ s^−1^ K^−1^ for N_2_. Generally, it is recommended to use He and H_2_ as carrier gases due to their high conductivity. TCD can be used with packed and capillary columns. After detection, the sample is not destroyed, which provides the possibility for further analyses (Gey 2015b).

*ECD* is mainly used to detect halogenated and sulfur-containing analysts (Gey 2015b). The field of operation is quite broad and focusing on the quantitative detection of polychlorinated biphenyls (Ballschmiter and Zell 1980), insecticides, and pesticides. Through a combination of dispersive liquid–liquid microextraction (DLLME) followed by derivatization and GC-ECD, chlorophenols could be detected in water sample (Fattahi et al. 2007). The detection of several pesticide residues (organochlorine, organophosphorus, organonitrogen, and pyrethroid) in honey can be measured via supercritical fluid extraction (SFE) followed by GC-ECD (Rissato et al. 2004). The ECD is assembled with an ionization chamber containing gas inflow and outflow, anode, cathode, and thin nickel foil coated with radioactive isotope ^63^Ni. The coated ^63^Ni foil serves as radioactive source (β-emitter). The β decay leads to the generation of primary electrons that can clash with N_2_ molecule of the carrier gas. Through this reaction, positively charged N_2_ molecules and free secondary electrons are produced. Applied voltage produces an electric field that moves secondary electrons to the anode, which results in a basic ionization flow. Halogenated and sulfur-containing analysts that have a high electron affinity can catch free electrons in the ECD and thereby reduce the ionization flow leading to a declined detector signal (Gey 2015b).

To identify and quantify gaseous components in the headspace of the cultivation device, mass spectrometry (MS) could be coupled to GC. Conversion and consumption of gaseous labeled substrates to gaseous end products could be elucidated via GC combined with an isotope ratio mass spectrometry (GC-CIRMS) (Martinez-Cruz et al. 2017). ^13^C metabolic flux analysis (^13^C-MFA), for instance, can be used to investigate metabolic flux distributions in multiple species simultaneously without any physical separation of cells. The metabolic fluxes for each species in a co-culture system are estimated directly from isotopic labeling of total biomass obtained using conventional mass spectrometry approaches such as GC-MS (Gebreselassie and Antoniewicz 2015). An isotopically labeled substrate is added to the culture, resulting in the incorporation of ^13^C atoms eventually into products. The contribution of a particular substrate to the formation of the end product can be quantified by applying this method. According to constant measurements of ^13^C-labeling, relative rates of substrate utilization can be investigated. This method is a useful tool for testing new pathways for the conversion of non-traditional feedstock (Gonzalez and Antoniewicz 2017). Sugar and biomass synthesis from CO_2_ of heterotrophic organism via non-native carbon fixation machinery was investigated by the following method (Antonovsky et al. 2016). Respectively to mass isotopologues distribution analysis, *Escherichia coli* BW25113 strain were cultured in a minimal media, either in the presence of a uniformly labeled ^13^C-pyruvate and unlabeled CO_2_ or in an inverse experimental set-up with isotopically labeled ^13^CO_2_ (Cambridge Isotope Laboratories) and a non-labeled pyruvate. Cells were harvested during exponential growth phase and lyophilized. The ratio of ^13^C/^12^C was determined using an elemental analyzer linked to a Micromass (Manchester, UK) Optima IR-MS (Antonovsky et al. 2016).

### Infrared sensors

Infrared (IR) sensors are available for CO_2_ and CH_4_ detection. Usually, for these sensors a wavelength in the NIR region is used (Renard et al. 1988; Holubar et al. 2002). Online IR sensors could be applied to track CO_2_ and CH_4_ production in the cultivation vessel.

## Discussion—quantification of gaseous substrates and products

The quantification of gaseous substrates and products is of immense interest—especially when working with anaerobes since many anaerobes can utilize gaseous substrate or produce gaseous products. Gaseous compound quantification can be based on weight increase if the consumption of applied gaseous substrates is associated with production of liquids (H_2_O method), pressure alteration, and gas flow, via GC or infrared spectroscopy. The H_2_O method can be seen as a cost-effective alternative to the GC, due to the fact that only a manometer and an analytical balance are needed to apply this method, whereas quantification via GC is a direct measurement technique that enables the analysis of the gaseous composition of a gas mixture. Due to the contamination risk of the sample, offline GC analysis is generally used as an end-point measurement. However, the H_2_O method can be used for indirect continuous gas production (Taubner and Rittmann 2016). Altering pressure in a cultivation device (closed batch or batch cultivation) could give indications about gas consumption or production. In open systems like bioreactor set-ups, either fed-batch or continuous mode, off-gas determinations could give insights in gas consumptions or product formation. When comparing all discussed flow meter devices, gas-type meters show the highest accuracy of 0.5% over the whole measuring range. However, it has the lowest maximum pressure and temperature range. All other flow meters can be applied at maximum pressures over 100 bar. Averaging pitot tube, orifice, and venturi mass flow meters can be operated in the broadest temperature range. The temperature range of vortex and Coriolis flow meter is also impressive, − 200 to 426 °C and − 240 to 426 °C. Low relative pressure loss during measurements was shown for averaging pitot tube, venturi, ultrasonic, and thermal flow meters. Middle relative pressure loss during measurements can occur when using Coriolis, vortex, flow nozzle, and orifice flow meters (Green and Perry 2008). A more precise technique to quantify gaseous substrates or products is employed by online GC and IR spectroscopy. Especially online GC and/or infrared sensors should be applied when aiming to retrieve information about actual substrate or product concentration in the bioreactor. The coupling of GCs toward IRMS equipment is possible and enables the detection of labeled compounds (Martinez-Cruz et al. 2017).

## Conclusions

Anaerobic microorganisms are highly diverse with respect to their natural distribution on Earth. Due to their anoxic lifestyle, they conquered specific terrestrial areas on Earth that provide restricted substrates diversity. Probably, this niche adaption leads to the great metabolic versatility that anaerobes possess. Their metabolic versatility makes anaerobes interesting candidates for the application as anaerobic microbial cell factories. Whenever cultivation of anaerobic microorganism in a biotechnological context is performed, it might be important to monitor microbial growth, viability, and substrate uptake and product formation kinetics. Under anaerobic conditions, cultivation, sampling procedures, and the determination of physiological characteristics of anaerobic microbial population have to be adapted. Those physiological characteristics are essential biotechnological variables and can be used to improve yield or productivity of an anaerobic culture. The determination of those characteristics in anaerobic cultivation systems may be addressed by using different techniques for sampling, measuring growth, viability, and substrate uptake and product formation kinetics. However, determining the appropriate method or combination of methods respecting cultivation conditions and the desired yield of information about the cultivated microorganisms is still sometimes challenging. This review gives a thorough guidance to be able to make a careful decision on which methods are suitable for the quantification of substrate uptake, growth, and production kinetics in anaerobic microbiology and biotechnology.

All presented advantages and disadvantages of the summarized methods should assist the reader to choose an appropriate measuring technique for their specific purpose whether for laboratory, pilot plant, or industrial plant scale. Assigning a method to laboratory, pilot plant, or industrial plant is more difficult as it seems and must be purposefully chosen for careful process analytical technology. Before ascribing a technique to a biological process in a cultivation vessel, whether laboratory, pilot plant, or industrial plant scale, the operation mode has to be specified since not every technique can be performed under each operation mode. Table 3 relates methods to cultivation modes. Table 4 gives an overview of all discussed methods and provides support for choosing proper methods for special purposes. All discussed methods are graded in four groups: connection to the cultivation vessel, costs, complexity, and how time-consuming the quantification is. This grading could support and improve the decision-making process, and which method under which conditions and bioreactor settings should be applied. This listing should give support to find the right method for the applied scale: laboratory, pilot plant, or industrial plant.

**Table 3:** Methods to investigate (A) growth and viability, (B) substrate uptake and product quantification under several cultivation modes

	Method		Cultivation system				Reference
				Bioreactor			
			Closed batch	Batch	Fed-batch	Continuous	
A							
Growth	Direct cell counting	Microscopic enumeration	✓	✓	✓	✓	(Brock 1983; Koch 2007)
		Membrane filter sampling technique					
		Counting chamber					(Talking et al. 2014)
		Electronic enumeration	✓	✓	✓	✓	(Kubitschek 1969)
		Fluorescence activated cell sorter (FACS)	✓	✓	✓	✓	(Thompson et al. 2015)
	Estimation	Colony counting	✓	✓	✓	✓	(Hungate 1969)
		Most probable number (MPN)					(Wagner et al. 2012; Koch 2007)
	Biomass	Dry weight	✓	✓	✓	✓	(Tisa et al. 1982; Guerrero et al. 1985)
		Wet weight					(Tisa et al. 1982; Troller 1989)
	Light scattering and sensors	Photometer/spectrophotometer	✓	✓	✓	✓	(Günther and Bergter 1971))
		Optical sensor					(Ulber et al. 2003; Vojinović et al. 2006; Ude et al. 2014)
		Fluorescence senor					(Coppella and Rao 1990; Farabegoli et al. 2003)
		Spectroscopic sensor (near-infrared spectroscopy (NIR), electrochemical impedance spectroscopy (EIS))					(Olsson and Nielsen 1997; Landgrebe et al. 2010)
							(Slouka et al. 2016)
	Modeling	State estimation (particle filter algorithm—offline and online techniques)		✓	✓	✓	(Kager et al. 2018)
		Estimation of volumetric mass bio-density (dielectric spectroscopy and a soft sensor based on first-principle elemental balances)		✓	✓	✓	(Ehgartner et al. 2014, 2017)
Live/dead	Staining and microscope/FACS	LIVE/DEAD BacLight® bacterial viability kit	✓	✓	✓	✓	(Moissl et al. 2003)
		LDS-FISH					(Savichtcheva et al. 2005)
		BONCAT					(Hatzenpichler et al. 2014)
		BONCAT-FISH					
		BONCAT-FACS					(Hatzenpichler et al. 2016)
		MAR					(Nielsen and Nielsen 2010)
	Molecular-based methods	PMA-qPCR	✓	✓	✓	✓	(Nocker and Camper 2009; Heise et al. 2016)
		DNase I/Proteinase K					(Villarreal et al. 2013)
	Physicochemical parameters	ATP	✓	✓	✓	✓	(Chung and Neethling 1988; Abelho 2005)
		IMC					(Braissant et al. 2010)
		Calorimetric chips					(Van Herwaarden 2005)
		Enthalpy assay					(Torres et al. 2004)
B							
Liquid substrate and product quantification	Chromatography and mass spectroscopy/detectors	LC-ESI-MS (structure of biomolecules, protein–ligand interaction, competitive consumption of 2 substrates)	✓	✓	✓	✓	(Zea et al. 2003)
		LC-MALDI-TOF-MS (enzyme)					(Johansson et al. 2014)
		HPLC (separation of amino acids, peptides, proteins, lipids, vitamins, organic acids, or bases)					(Nollet and Toldrá 2012)
		HPLC-UV (formic, acetic, butyric, propionic acid)	✓	✓	✓	✓	(Wagner et al. 2011)
		HPLC-fluorescence (dissolved free taurine and amino acids)	✓	✓	✓	✓	(Clifford et al. 2017)
		HPLC-MALDI-TOF-MS (glycerol dialkyl glycerol tetraethers)	✓	✓	✓	✓	(Hopmans et al. 2000)
		UPLC (e.g., quinone)	✓	✓	✓	✓	(Hanif et al. 2012)
		UPLC-UV-ESI-MS/MS (e.g., heterocyclic amines (PhIP))	✓	✓	✓	✓	(Fekry et al. 2015)
	Spectroscopy	NIR (exopolysaccharide lactic acid, lactose, linoleic acid, oleic acid, and ammonia)		✓	✓	✓	(Acedo et al. 2002; Tiwari et al. 2013)
		MIR (e.g., methanol)		✓	✓	✓	(Schenk et al. 2007)
		Raman (dextrins, maltotriose, maltose, glucose lactate, ethanol, formate, acetate, and phenylalanine)		✓	✓	✓	(Shaw et al. 1999; Lee et al. 2004; Gray et al. 2013)
	Assay	ELISA/assay kits (starch, mono-, di-polysaccharides, alcohols, and organic acids)	✓	✓	✓	✓	(Neuhaus et al. 2015)
	Stable-isotope probing (SIP)	Isotope ratio mass spectroscopy (IRMS)	✓	✓	✓	✓	(Antonovsky et al. 2016)
		DNA-SIP or RNA-SIP (isotopes—^13^C, ^14^C, ^15^N, ^18^O, ^3^H, ^32/33^P,^35^S)	✓	✓	✓	✓	(Radajewski et al. 2000; Manefield et al. 2002)
		Single cell level: FISH-MAR	✓	✓	✓	✓	(Lee et al. 1999; Vila et al. 2004)
		FISH-SIMS					(Biddle et al. 2006; Musat et al. 2012)
		FISH-Raman	✓	✓	✓	✓	(Haider et al. 2010)
		NanoSIMS:	✓	✓	✓	✓	(Lechene et al. 2006)
		SIMSISH	✓	✓	✓	✓	(Amann and Fuchs 2008)
		EL-FISH	✓	✓	✓	✓	(Behrens et al. 2008)
		HISH-SIMS	✓	✓	✓	✓	(Halm et al. 2009)
		PLFA-SIP (proteins or phospholipid fatty acids)	✓	✓	✓	✓	(Middelburg et al. 2000)
Gaseous substrate and product quantification	Physical quantity	Gravimetric determination	✓	✓	✓	✓	(Taubner and Rittmann 2016)
		Volumetric based determination		✓	✓	✓	(Green and Perry 2008)
		Mass flow meter (Coriolis and capillary thermal mass flow meter)		✓	✓	✓	(Green and Perry 2008)
		Velocity flow meter (ultrasonic and vortex flow meter)		✓	✓	✓	(Green and Perry 2008)
		Differential pressure flow meter (orifice plate, flow nozzles, venturi, averaging pitot tube flow meter)		✓	✓	✓	(Green and Perry 2008)
		Positive displacement meter (drum-type gas meter)		✓	✓	✓	(Ritter GmbH, Bochum, Germany)
		Pressure alteration (pressure changes in the cultivation vessel)	✓	✓	✓	✓	(Follonier et al. 2012; Keymer et al. 2013; Taubner and Rittmann 2016)
	Chromatography and detectors and spectroscopy	GC-FID (most carbonic compounds; He, NH_3_, CO, CO_2_, H_2_S, H_2_O, O_2_, N_2,_ N_2_O, NO, NO_2_ give little or no response)	✓	✓	✓	✓	(McNair and Miller 2011)
		GC-TCD (H_2_, N_2_, O_2_, CO, CH_4_, and CO_2_)	✓	✓	✓	✓	(Gey 2015b)
		GC-ECD (halogenated and sulfur-containing analysts)	✓	✓	✓	✓	(Gey 2015b)
		GC-IRMS (^13^C/^12^C)	✓	✓	✓	✓	(Martinez-Cruz et al. 2017)
		NIR (CO_2_ and CH_4_)		✓	✓	✓	(Renard et al. 1988; Holubar et al. 2002)

**Table 4:**
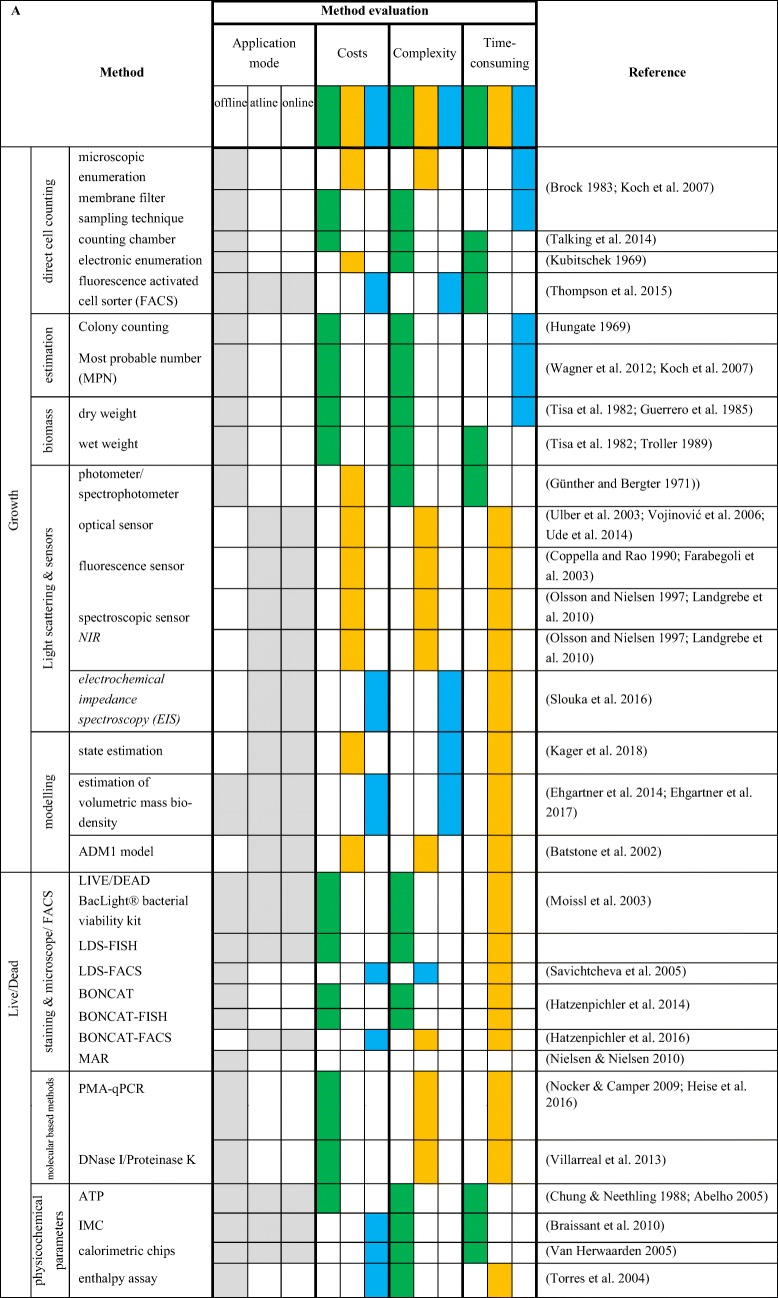

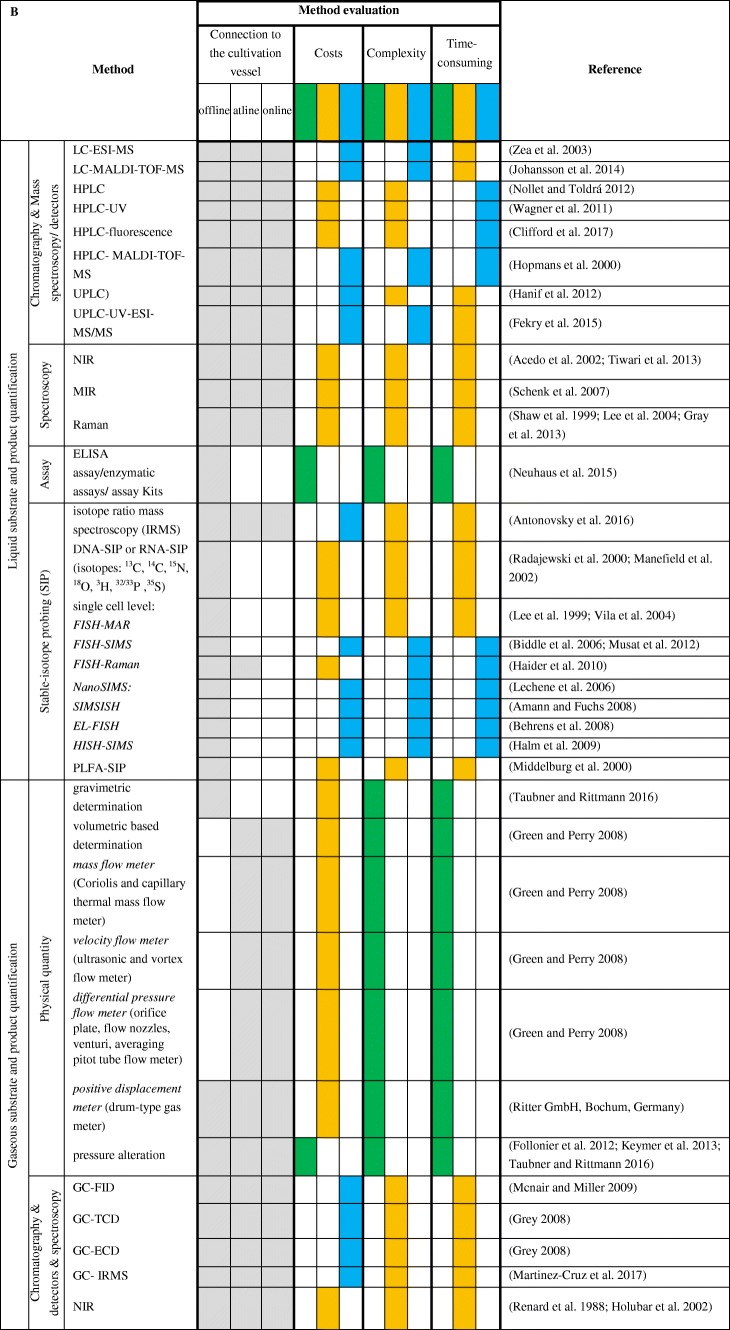
Methods are categorized through the application mode, costs, complexity of the method, and analysis time (A) Growth and viability, (B) substrate uptake and product quantification techniques. Gray: usable; green: low; orange: middle; blue: high

## Abbreviations

VFA: volatile fatty acid
H_2_: molecular hydrogen
CO_2_: carbon dioxide
CH_4_: methane
CO: carbon monoxide
O_2_: molecular oxygen
N_2_: molecular nitrogen
CO_2_-BMP: CO_2_-based biological methane production
*s*: substrate
*t*: time
*q*: specific substrate consumption rate
*Y*_( *x*/ *s*)_: biomass and substrate yield coefficient
*X*: biomass
*μ*: specific growth rate
ORP: oxidation-reduction potential
MPN: most probable number
FACS: fluorescence activated cell sorting
OD: optical density
NIR: near-infrared region
MIR: mid-infrared region
FIR: far infrared region
EIS: electrochemical impedance spectroscopy
ADM1: anaerobic digestion model No. 1
ATP: adenosine triphosphate
MAR: microautoradiography
BONCAT: bio-orthogonal non-canonical amino acid tagging
DNA: deoxyribonucleic acid
PMA: propidium monoazide
RNA: ribonucleic acid
mRNA: messenger ribonucleic acid
rRNA: ribosomal ribonucleic acid
IMC: isothermal calorimetry
LC: liquid chromatography
HPLC: high performance liquid chromatography
UPLC: ultra performance liquid chromatography
MS: mass spectrometry
IRMS: isotope ratio mass spectrometry
ESI: electrospray ionization
CI: chemical ionization
FI: field ionization
FD: field desorption
FAB: fast atom bombardment ionization
MALDI: matrix-assisted laser desorption/ionization
EI: electron ionization
APCI: atmospheric pressure chemical ionization
APPI: atmospheric pressure photoionization
MS/MS: magnetic sector mass analyzer
QMS: quadrupole mass analyzer
TOF: time-of-flight mass analyzer
IT: trapped-ion mass analyzers
QIT: quadrupole ion trap mass analyzer
PMT: photomultiplier tube detector
EMT: electron multiplier tube detector
FC: Faraday cup detector
SIP: stable isotope probing
PLFA-SIP: phospholipid fatty acids -SIP
Bq: Becquerel (radioactive decay s^−1^)
Sv: Sieverts (J kg^−1^)
GC: gas chromatograph
WCOT: wall coated open tubular column
PLOT: porous layer open tubular column
SCOTT: support coated open tubular column
FID: flame ionization detector
TCD: thermal conductivity detector
ECD: electron capture detector
